# Deployment of *Brassica carinata* A. Braun Derived *Brassica juncea* (L.) Czern. Lines for Improving Heterosis and Water Use Efficiency Under Water Deficit Stress Conditions

**DOI:** 10.3389/fpls.2021.765645

**Published:** 2021-11-25

**Authors:** Omkar Maharudra Limbalkar, Rajendra Singh, Parvesh Kumar, Joghee Nanjundan, Chiter Mal Parihar, Prashant Vasisth, Devendra Kumar Yadava, Viswanathan Chinnusamy, Naveen Singh

**Affiliations:** ^1^Division of Genetics, Indian Council of Agricultural Research-Indian Agricultural Research Institute, New Delhi, India; ^2^Indian Council of Agricultural Research-Indian Agricultural Research Institute Regional Station, Wellington, India; ^3^Division of Agronomy, Indian Council of Agricultural Research-Indian Agricultural Research Institute, New Delhi, India; ^4^ADG (Seeds), Indian Council of Agricultural Research, New Delhi, India; ^5^Division of Plant Physiology, Indian Council of Agricultural Research-Indian Agricultural Research Institute, New Delhi, India

**Keywords:** *Brassica juncea*, interspecific hybridization, gene effects, heterosis, water use efficiency, drought tolerance

## Abstract

Among *Brassica* species, Ethiopian mustard (*Brassica carinata* A. Braun) is known to tolerate most abiotic stresses, including drought. Drought caused by low and erratic rainfall in semi-arid regions consistently challenges rapeseed mustard productivity. Development of *B. carinata*-derived lines (CDLs) in *Brassica juncea* (L.) Czern. nuclear background, carrying genomic segments from *B. carinata*, are expected to tolerate moisture deficit stress conditions. The present study was, thus, aimed to establish the phenomenon “heterosis” for drought tolerance and water use efficiency by evaluating 105 hybrids developed from intermating 15 CDLs in half diallel fashion. Data on 17 seed yield and yield contributing traits were recorded under two different environments, *viz*., irrigated and rainfed conditions. Traits under study were found to be governed by both additive and non-additive types of gene action. Average degree of dominance was higher (>2) for yield and yield contributing traits, *viz*., secondary branches/plant, point to first siliqua on main shoot, total siliquae/plant, 1,000-seed weight, seed yield/plant, biological yield, harvest index, and seed yield/hectare under rainfed conditions, clearly indicating that higher productivity under drought conditions can be realised through the development of hybrids. Out of 15, highly significant general combining ability (GCA) effects for seven CDLs were observed under rainfed condition. Furthermore, nine and six hybrids expressed highly significant specific combining ability (SCA) effects and > 50% heterobeltiosis for yield contributing traits under rainfed and irrigated conditions, respectively. Water use efficiency (WUE) of parental CDLs and hybrids varied from 2.05 to 2.57 kg m^–3^ under rainfed, while 1.10 to 1.28 kg m^–3^ under irrigated conditions. Hybrids expressed higher WUE than parental lines under both water regimes. Furthermore, selection indices such as drought tolerance index (DTI) and mean relative performance (MRP) were identified to be efficient in the selection of productive CDLs and hybrids under drought conditions. Nine hybrids, identified as highly productive in the present study, can further be exploited for improving the yield of Indian mustard in drought-prone areas. Usefulness of interspecific hybridisation in the development of *B. carinata*-derived *B. juncea* lines for improving heterosis and WUE is, thus, well demonstrated through the present study.

## Introduction

Rapeseed-mustard group is the second most important oilseed crop in the world after soybean with a production of 69.08 million metric tons and contributed 11.89% to global oilseed production (580.51 million metric tons) in 2019–2020 (Anonymous, 2021). India is the third-largest producer of rapeseed-mustard group of crops followed by Canada and China with a total production of 7.4 million metric tons from a 7.1 mha area (Anonymous, 2021). Indian mustard, *Brassica juncea* (L.) Czern. (AABB; 2n = 36), is an amphidiploid species that originated from a spontaneous interspecific hybridisation of *Brassica rapa* L. (AA, 2n = 20) and *B. nigra* L. (BB, 2n = 16). It is a widely cultivated species among the rapeseed-mustard group of crops accounting for more than 90% of its total acreage in India. Being a pivotal crop of the Indian oilseed sector, extensive efforts have been made to improve its seed and oil yields to achieve national self-sufficiency in edible oils. Despite that, a huge amount of edible oil is being imported to meet the requirements for domestic consumption. The Solvent Extractors’ Association of India reported that the import of edible oil rises to 23 million metric tons, costing more than 11 thousand million dollars in 2020 in the country, which accounts for more than 60% of the total edible oil demand.

To achieve self-sufficiency in edible oils, there is an urgent need to improve the productivity of Indian mustard. However, narrow genetic base of working gene pool is a major impediment in the improvement of this species (Chauhan et al., 2011). Most of the released Indian varieties are either derived directly from the cultivar Varuna or its derivatives (Singh and Chauhan, 2010; Chauhan et al., 2011). The yield potential of these varieties is about 4 t/ha; however, the national average yield is hovering around 1.5 t/ha. The stochastic production patterns are largely attributed to its susceptibility to different biotic and abiotic factors (Sharma et al., 2018). Indian mustard group of crops is mainly cultivated by marginal and small-farmers, wherein about 25% of the rapeseed-mustard area is rainfed, causing critical yield losses particularly in the drought-prone areas of eastern and western parts of the country (Sharma et al., 2018). Most of the cultivars of Indian mustard are susceptible to drought and encounter severe yield losses up to 94% (Sharma and Kumar, 1989). Although few varieties of Indian mustard have been released for commercial cultivation in rainfed areas of the country (Chauhan et al., 2011), these varieties failed to occupy sufficiently large area in the drought prone regions. This indicates that target environments still demand a better level of tolerance in future varieties. This also calls for the urgent development of high-yielding water use efficient genotypes and hybrids which can withstand drought conditions to minimise the yield losses, improve productivity and stabilise mustard production.

Relatives of *B. juncea* [*Sinapis alba* (Warwick, 1993), *Arabidopsis thaliana* (Kasuga et al., 1999); *B. rapa* (Seo et al., 2010), *B. napus* (Dalal et al., 2009; Fletcher et al., 2015), *B. carinata* (Malik, 1990)] harbour many useful traits, such as tolerance to drought, salinity, and cold. These could be used to incorporate abiotic stress tolerance in present-day cultivars (Warwick, 1993). Furthermore, wide hybridisation has been considered a novel method for generating selectable genetic variability in cultivated species (Prakash, 1973; Inomata, 1997).

*Brassica carinata* (BBCC; 2n = 34), an important oilseed crop of Ethiopian origin, is being cultivated in northeast Africa, parts of Canada, France, Spain, Australia, China, and India. Although this species has tall plant stature, poor seed characteristics, and takes longer to mature (Supplementary Table 1), when compared to *B. juncea*, it possesses many agronomically desirable traits including resistance or tolerance to most of the abiotic and biotic stresses like drought, heat, aphid, Sclerotinia rot, white rust, Alternaria black spot, blackleg, and powdery mildew (Raman et al., 2017; Thakur et al., 2019), which are otherwise eliminated from the predominantly cultivated *Brassica* species during domestication. Therefore, efforts toward interspecific hybridisation were made in the past to transfer desirable traits from *B. carinata* to cultivated *Brassica* species *viz*., *B. rapa* (Jiang et al., 2007; Choudhary et al., 2008), *B. napus* (Navabi et al., 2011; Sheikh et al., 2014), and *B. juncea* (Getinet et al., 1994; Sheikh et al., 2014; Singh K. H. et al., 2015). *B*. *carinata* is known to be the most drought tolerant among cultivated *Brassica* species as it develops sufficient biomass and seed yield even under the water deficit stress conditions. However, rainfall or irrigation leads to higher biomass accumulation, lodging of crop and, thus, reduction in seed size, oil content, and other yield related traits in this species (Thakur et al., 2019).

Keeping this in view, efforts were made at the Indian Council of Agricultural Research-Indian Agricultural Research Institute (ICAR-IARI), New Delhi to enrich the genome of *B. juncea* with *B. carinata*, through the development of *B. carinata*-derived *B. juncea* lines (CDLs), for improving their ability to withstand drought conditions. Though inter-subgenomic heterosis were reported in hybrids of *B. rapa* and *B. carinata*-derived introgression lines with *B. juncea* accessions (Wei et al., 2016), the present study aimed to use *B. carinata*-derived *B. juncea* lines for improving heterosis and water use efficiency (WUE) under water deficit stress conditions. To the best of our knowledge, this is the first case where diverse CDLs were developed and deployed for improving drought tolerance.

Hybrid breeding involving diverse parental lines is considered one of the most viable options for breaking the yield barrier (Singh N. et al., 2015). Involvement of alien introgression lines in hybrid development enables stretching of genetic diversity among parental lines required for the expression of higher heterosis, complementation of favorable novel alleles from both the introgressed parents in the F_1_ generation, and, thus, improved ability of hybrids to withstand stress. Information on gene action, their relative contribution to the genetic variance, and estimation of general and specific combining ability effects (GCA and SCA) under water deficit stress conditions are pre-requisite for the identification of best parental combinations enabling the development of high yielding drought tolerant mustard hybrids (Panhwar et al., 2008; Khan et al., 2009). Several selection indices have also been suggested for the identification of water use efficient genotypes and cross combinations in crop plants, including mustard (Fischer and Maurer, 1978; Rosielle and Hamblin, 1981; Clarke et al., 1984; Fernandez, 1992; Huang, 2000). Despite this, this information was not much helpful in mitigating the ill-effect imposed by drought conditions in Indian mustard, primarily due to inherent genetic ceiling in this species.

*Brassica juncea* CDLs possessing useful genomic segment(s) from *B. carinata*, developed at the Indian Council of Agricultural Research – Indian Agricultural Research Institute (ICAR-IARI), New Delhi, shall be useful in the identification of high yielding drought tolerant parental lines and hybrids. Furthermore, information generated on gene action and degree of dominance for yield contributing traits and WUE under moisture deficit stress condition in *B. carinata* derived lines and hybrids shall help in devising future breeding strategies. The present study was, therefore, conducted to (i) determine the type of gene action, combining ability of parental lines and heterobeltiosis in the hybrids developed by using CDLs, (ii) assess the WUE of CDLs and their hybrids, and (iii) identify water use efficient genotypes and cross combinations.

## Materials and Methods

### Selection of *Brassica carinata* Derived *Brassica juncea* Lines

**FIGURE 1:**
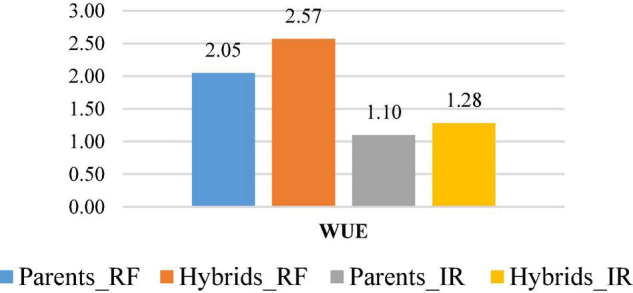
WUE (kg m^–3^) in *B. carinata* derived *B. juncea* lines (Parents) and their hybrids under rainfed (RF) and irrigated (IR) conditions.

**FIGURE 2:**
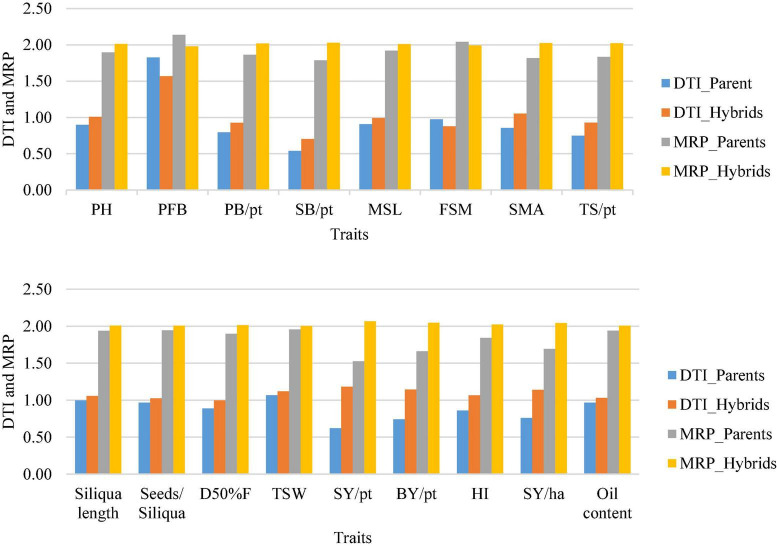
The drought tolerant index (DTI) and mean relative performance (MRP) observed on seventeen traits in hybrids along with parents grown in rainfed and irrigated conditions. PH, plant height; PFB, point to first branch; PB/pt, primary branches/plant, SB/pt, secondary branches/plant; MSL, main shoot length; FSM, point to first siliqua on main shoot; SMS, number of siliquae on main shoot; TS/pt, total siliquae/plant; D50%F, day to 50% flowering; TSW, thousand seed weight; SY/pt, seed yield/plant; BY/pt, biological yield/plant; HI, harvest index; SY/ha, seed yield kg per hectare.

A set of F_2_ populations was generated from crossing *B. juncea* genotypes (DRMRIJ 31, Pusa Mustard 30 and Pusa Agrani) with *B. carinata* accessions (BC-4, BC-5, and BC-12). Within each of these F_2_ populations, plants were selected on the basis of phenotype and were inter-mated in pairs – a method commonly called biparental mating. Phenotypic selection in the subsequent filial generations led to the development of *B. carinata*-derived *B. juncea* lines (CDLs). These cytologically stable homozygous CDLs possess 18 bivalents (2n = 36), indicating its similarity to *B. juncea* (upplementary Figures 1, 2). These CDLs, in F_6_ generation, were evaluated in two rows of 4-m length plots under both irrigated and rainfed field condition in the Division of Genetics, ICAR- IARI, New Delhi during 2018–2019 *rabi* season. To select CDLs differing in metric traits and response to moisture deficit stress, row-row and plant–plant spacing were kept at 30 and 15 cm, respectively (data not presented). One row was left blank before and after every plot such that plot–plot and row–row in a plot were separated by a spacing of 60 and 30 cm, respectively. The experiment was raised in augmented design both under irrigated and rainfed conditions. No irrigation was applied in the rainfed plot, while two irrigations, each of 50 mm depth, were applied in irrigated plots at 45 and 90 days after sowing (DAS). All recommended agronomic practices were adopted for raising the crop. Based on drought tolerance indices, 15 CDLs differing for various morpho-physiological traits and phenotypical and cytological resemblance to *B. juncea* (Supplementary Table 1 and upplementary Figures 1, 2) were selected for generating hybrids.

### Generation of Crosses

During 2019, 15 diverse CDLs differing for morpho-physiological traits were intermated in half diallel design (excluding reciprocals) to generate seeds of 105 F_1_ hybrids in ICAR-IARI, Regional Station, Wellington, Tamil Nadu, India in the off-season.

### Evaluation of Hybrids and Their Parental Lines

During *rabi* 2019–2020 season, the set of 105 F_1_ hybrids along with 15 parental CDLs was raised under both rainfed and irrigated conditions in the research field of Division of Genetics, ICAR-IARI, New Delhi. Two recommended irrigations of 50 mm depth each were applied at 45 and 90 DAS to the irrigated plots, while no irrigation was given to rainfed plots. Hybrids along with parents were raised in a randomised complete block design (RCBD) with three replications. Each plot consisted of a paired row of 4 m in length. Row to row and plant to plant distance were kept 30 and 15 cm, respectively. One row of 30 cm was left blank before and after every pair-row plot such that plot–plot and row–row in a plot were distanced at 60 and 30 cm, respectively. Recommended agronomical practices and plant protection measures were adopted for raising the crop.

Observations were recorded on 17 quantitative traits, *viz*., plant height (cm), point to first branch (cm), number of primary branches, number of secondary branches, main shoot length (cm), point to first siliqua on main shoot (cm), number of siliquae on main shoot, total siliquae per plant, siliqua length (cm), seeds per siliqua, days to 50% flowering, 1,000-seed weight (g), seed yield per plant (g), biological yield per plant (g), harvest index, seed yield (kg/ha), and oil content. The data were recorded on five randomly selected competitive plants from all the plots in each replication, except for seed yield per plot (g) and days to 50% flowering where observations were recorded on plot basis. Biological yield was recorded at physiological maturity after drying the harvested plants. Data on plot yield (g) were used to calculate seed yield (kg/ha).

### Assessment of Drought Tolerance

Data were recorded on 17 metric traits under both irrigated and rainfed conditions. Mean values of these traits were used to analyse drought susceptibility index (DSI; Fischer and Maurer, 1978), drought tolerance index (DTI; Fernandez, 1992), tolerance index (TOL; Rosielle and Hamblin, 1981), and mean relative performance (MRP) for the CDLs taken in this study (Raman et al., 2012).

Water-use efficiency (WUE) for parents and hybrids were calculated from the below given equation:

WUE [kg ha^–1^mm^–1^] = Seed Yield (kgha^–1^)/Water received from irrigation and rainfall (mm)

and WUE [kg m^–3^] = 0.1 × WUE [kg ha^–1^mm^–1^].

The effective rainfall (between October 2019 and February 2020) was calculated by using Cropwat Version 8.0 by USDA SCS method. Cropwat is a computer-based decision support tool developed by the Food and Agriculture Organization (FAO) of the United Nations to estimate effective rainfall based on climate and soil data. The maximum and minimum temperatures and rainfall (mm) recorded during 2019–2020 crop seasons from ICAR-IARI meteorological observatory, located within 500 m distance from the experimental site, is presented in Supplementary Figure 3.

### Statistical and Genetic Analyses

The mean values obtained from 17 different quantitative traits were subjected to analysis of mean and variance. Data obtained from 105 F_1_ and 15 parental CDLs were subjected to Griffing’s method II with a fixed model (model I) to determine the combining abilities (Griffing, 1956). The mathematical model for *ijk*th observation is as follows:


Yij=μ+g+ig+js+ijb+keijk

where *Y*_*ij*_ is the mean of *i* × *j^th^* genotype over *k*; μ is general population mean; *g*_*i*_ and *g*_*j*_ is the general combining ability effects of *i^th^* and *j^th^* parent, respectively; s*_*ij*_* is the specific combining ability effects of cross involving *i^th^* and *j^th^* parent, respectively; and *b*_*k*_ is the replication effect; and e*_*ijk*_* is the residual error.

Heterobeltiosis expressed in the hybrids was calculated by using mean values of respective F_1_ and its better parent (BP) following standard formula (Coffman, 1933). The narrow-sense heritability (*h*^2^) was calculated by using the method suggested by Mather and Jinks (1982). Analysis of variance and estimation of degree of dominance, heritability, and combining ability were done following the method suggested by Griffing (1956) using Windostat Version 9.1. The degree of dominance was estimated from variance due to dominance (σ^2^_*D*_) and variance due to additive (σ^2^_*A*_) gene action by using the formula [{(2 × σ^2^_D_)/σ^2^_A_}^1/2^] given by Comstock and Robinson (1948).

## Results

Sufficient phenotypic variation was observed for the seed yield and its contributing traits under both rainfed and irrigated conditions. Most of the traits were normally distributed except days to 50% flowering (Supplementary Figure 4). There were significant differences (*p* < 0.001) among the parents and hybrids for all the recorded 17 traits, *viz*., plant height, point to the first branch, number of primary branches per plant, number of secondary branches per plant, main shoot length, point to the first siliqua on the main shoot, number of siliquae on the main shoot, total number of siliquae per plant, days to 50% flowering, 1,000-seed weight (g), seed yield per plant (g), biological yield per plant (g), harvest index, seed yield (kg/ha), and oil content (%) under irrigated (Table 1) and rainfed (Table 2) conditions. The mean squares of parents vs. hybrids under irrigated condition were found to be significant for all traits except point to first siliqua on the main shoot while under the rainfed condition, it was also significant for most of the traits except point to the first siliqua on the main shoot, seeds per siliqua, 1,000-seed weight, and oil content. Results also exhibited a significant mean sum of squares due to hybrids for all the traits under both rainfed and irrigated conditions (Tables 1, 2).

**TABLE 1:** Analysis of variance for parents and hybrids generated in half diallel design for studying different yield contributing traits under irrigated conditions.

	Sources of variations							
Traits	Replications	Female:male	Parents	Hybrids	Parents Vs. Hybrids	Error	Mean	CV (%)
	d.f. = 2	119	14	104	1	238		
PH	53.50	764.78**	1047.12**	660.99**	7605.9**	140.32	230.07	5.15
	(0.38)	(5.45)	(7.46)	(4.71)	(54.2)			
PFB	12.47	717.21**	685.04**	726.95**	155.17**	5.72	32.34	7.39
	(2.18)	(125.38)	(119.76)	(127.08)	(27.12)			
PB/pt	0.64	3.66**	2.33**	3.71**	17.17**	0.92	7.60	12.65
	(0.69)	(3.96)	(2.52)	(4.01)	(18.56)			
SB/pt	3.81	51.78**	34.41**	50.68**	409.29**	1.71	17.89	7.32
	(2.23)	(30.2)	(20.07)	(29.56)	(238.68)			
MSL	4.41	185.65**	104.47**	190.97**	768.96**	37.81	79.73	7.71
	(0.12)	(4.91)	(2.76)	(5.05)	(20.33)			
FSM	1.43	40.35**	28.12**	42.37**	1.91	0.78	8.37	10.56
	(1.84)	(51.64)	(35.98)	(54.22)	(2.45)			
SMS	15.27	202.08**	121.12**	202.83**	1258.01**	38.32	53.59	11.55
	(0.4)	(5.27)	(3.16)	(5.29)	(32.82)			
TS/pt	271.49	79803.8**	29008.97**	84878.04**	263209.97**	1443.53	529.02	7.18
	(0.19)	(55.28)	(20.09)	(58.79)	(182.33)			
Siliqua length	0.127	0.517**	0.697**	0.49**	0.781**	0.062	4.15	5.99
	(2.06)	(8.37)	(11.27)	(7.93)	(12.64)			
Seeds/Siliqua	0.55	7.5**	8.33**	7.17**	30.63**	1.55	15.89	7.85
	(0.35)	(4.83)	(5.35)	(4.61)	(19.69)			
D50%F	3.13	107.05**	116.8**	98.61**	848.42**	12.69	56.73	6.28
	(0.25)	(8.43)	(9.19)	(7.76)	(66.81)			
TSW	0.17	0.87**	0.802**	0.851**	3.46**	0.079	3.96	7.1
	(2.13)	(10.94)	(10.12)	(10.73)	(43.68)			
SY/pt	2.51	302.77**	50.39**	303.21**	3790.15**	12.63	28.94	12.28
	(0.2)	(23.97)	(3.98)	(24)	(300.02)			
BY/pt	44.22	6404.17**	1012.63**	6632.03**	58188.069**	183.91	151.76	8.94
	(0.24)	(34.82)	(5.5)	(36.06)	(316.38)			
HI	0.47	33.15**	31.39**	32.4**	135.77**	1.3	19.00	6.02
	(0.36)	(25.38)	(24.03)	(24.8)	(103.94)			
SY/ha	86779.4	1071068**	1473003**	1018435**	917764.3**	39013.3	2670.5	7.39
	(2.22)	(27.45)	(37.75)	(26.10)	(23.52)			
Oil content	7.06	17.77**	27.27**	15.26**	146**	2.4	33.32	4.66
	(2.93)	(7.37)	(11.31)	(6.33)	(60.57)			

*d.f., degrees of freedom; PH, plant height; PFB, point to first branch; PB/pt, primary branches/plant; SB/pt, secondary branches/plant; MSL, main shoot length; FSM, point to first siliqua on main shoot; SMS, number of siliquae on main shoot; TS/pt, total siliquae/plant; D50%F, days to 50% flowering; TSW, thousand seed weight; SY/pt, seed yield/plant; BY/pt, biological yield/plant; HI, harvest index; SY/ha, seed yield kg per hectare.*

**Significant at p = 0.01 and *significant at p = 0.05.

**TABLE 2:** Analysis of variance for parents and hybrids generated in half diallel design for studying different yield contributing traits under rainfed conditions.

	Sources of variations							
Traits	Replications	Female:male	Parents	Hybrids	Parents Vs. Hybrids	Error	Mean	CV (%)
Df	d.f. = 2	119	14	104	1	238		
PH	39.22	729.2**	1070.57**	626.1**	6672.91**	213.8	228.26	6.4
	(0.18)	(3.41)	(5)	(2.92)	(31.21)			
PFB	11.8	566.33**	528.52**	568.31**	889.64**	22.04	48.45	10.02
	(0.54)	(25.69)	(23.97)	(25.78)	(40.35)			
PB/pt	0.194	2.83**	4.78**	2.5**	8.5**	0.318	6.90	8.16
	(0.61)	(8.88)	(15.04)	(7.88)	(26.72)			
SB/pt	0.2	26.24**	8.47**	28.68**	21.34**	1.52	12.10	10.66
	(0.13)	(17.22)	(5.56)	(18.82)	(14)			
MSL	1.24	150.71**	240.6**	137.29**	287.48**	28.37	78.12	6.82
	(0.04)	(5.31)	(8.47)	(4.83)	(10.13)			
FSM	0.15	24.57**	19.68**	25.46**	0.99	0.298	7.37	7.42
	(0.53)	(82.22)	(65.84)	(85.17)	(3.33)			
SMS	2.34	161.89**	171.46**	150.41**	1221.35**	36.25	54.84	10.98
	(0.06)	(4.47)	(4.72)	(4.14)	(33.68)			
TS/pt	1608.67	44551.2**	28892.99**	46996.99**	9402.96**	859.5	470.32	6.23
	(1.87)	(51.83)	(33.61)	(54.67)	(10.94)			
Siliqua length	0.11	0.67**	1.38**	0.57**	0.9**	0.072	4.33	6.23
	(1.52)	(9.3)	(19.02)	(7.96)	(12.48)			
Seeds/Siliqua	0.33	8.03**	10.81**	7.73**	0.38	1.47	16.06	7.59
	(0.23)	(5.44)	(7.32)	(5.23)	(0.26)			
D50F	3.811	72.66**	57.13**	74.1**	140.95**	9.79	55.61	6.71
	(0.39)	(7.42)	(5.83)	(7.56)	(14.38)			
TSW	0.58	1.14**	0.97**	1.16**	0.46	0.144	4.40	8.60
	(4.06)	(7.9)	(6.73)	(8.09)	(3.25)			
SY/pt	6.35	270.52**	44.47**	288.31**	1584.87**	9.39	31.34	9.77
	(0.68)	(28.8)	(4.73)	(30.69)	(168.73)			
BY/pt	87.68	5618.51**	757.68**	6146.71**	18737.27**	149.49	163.35	7.46
	(0.59)	(37.58)	(5.06)	(41.11)	(125.33)			
HI	0.053	81.06**	34.29**	87.13**	104.95**	0.99	19.57	5.10
	(0.05)	(81.11)	(34.31)	(87.18)	(105.01)			
SY/ha	81486	1399416**	2141042**	1310111**	304341.8**	39844.4	2819.37	7.08
	(2.04)	(35.12)	(53.73)	(32.88)	(7.63)			
Oil content	0.68	16.79**	24.12**	15.93**	3.93	2.18	34.03	4.34
	(0.31)	(7.69)	(11.04)	(7.29)	(1.801)			

*d.f., degrees of freedom; PH, plant height; PFB, point to first branch; PB/pt, primary branches/plant; SB/pt, secondary branches/plant; MSL, main shoot length; FSM, point to first siliqua on main shoot; SMS, total siliqua on main shoot, TS/pt, total siliqua/plant; D50%F, days to 50% flowering; TSW, thousand seed weight; SY/pt, seed yield/plant; BY/pt, biological yield/plant; HI, harvest index; SY/ha, seed yield kg per hectare.*

**Significant at p = 0.01 and *significant at p = 0.05.

The variance due to specific combining ability (σ^2^sca) were higher than general combining ability variance (σ^2^gca) for traits, *viz*., point to the first branch, number of primary branches, number of secondary branches, main shoot length, point to first siliqua on the main shoot, number of siliquae on the main shoot, total siliquae per plant, days to 50% flowering, 1,000-seed weight (g), seed yield per plant (g), biological yield per plant (g), harvest index, seed yield (kg/ha), and oil content (%) and, thus, the average degree of dominance for these traits were observed to be more than one (Table 3). For siliqua length and seeds per siliqua under both irrigated and rainfed conditions, the average degree of dominance was less than unity. For plant height, σ^2^sca > σ^2^gca and the degree of dominance was found to be more than one under irrigated, whereas σ^2^gca > σ^2^sca and degree of dominance was less than unity under rainfed condition.

**TABLE 3:** Estimates of genetic components of variance for seventeen characters in *B. carinata*-derived *B. juncea* lines (CDLs) and their hybrids evaluated under irrigated and rainfed conditions.

Components of variance		σ^2^_gca_	σ^2^_sca_	σ^2^_gca_/σ^2^_*sca*_	σ^2^_A_	σ^2^_D_	Average degree of dominance	*h* ^2^
Plant height (cm)	IR	69.75	77.81	0.90	139.50	77.81	1.06	0.52
	RF	64.27	49.03	1.31	128.53	49.03	0.87	0.52
Point to first branch (cm)	IR	46.93	162.34	0.29	93.86	162.34	1.86	0.36
	RF	36.22	123.53	0.29	72.44	123.53	1.85	0.36
Primary branches/plant	IR	0.19	0.59	0.33	0.39	0.59	1.75	0.30
	RF	0.18	0.54	0.33	0.35	0.54	1.75	0.35
Secondary branches/plant	IR	0.88	16.93	0.05	1.75	16.93	4.40	0.09
	RF	1.03	6.99	0.15	2.07	6.99	2.60	0.22
Main shoot length (cm)	IR	12.57	27.35	0.46	25.14	27.35	1.48	0.38
	RF	7.22	29.84	0.24	14.44	29.84	2.03	0.27
Point to first siliqua on main shoot (cm)	IR	1.90	10.65	0.18	3.79	10.65	2.37	0.25
	RF	0.57	7.86	0.07	1.14	7.86	3.71	0.13
Total siliqua on main shoot	IR	12.54	33.43	0.38	25.08	33.43	1.63	0.35
	RF	7.67	30.06	0.26	15.34	30.06	1.98	0.27
Total number of siliqua/plant	IR	3917.17	20723.84	0.19	7834.34	20723.84	2.30	0.27
	RF	1343.07	13461.46	0.10	2686.14	13461.46	3.17	0.16
Siliqua length (cm)	IR	0.06	0.03	1.85	0.12	0.03	0.74	0.69
	RF	0.09	0.03	2.50	0.17	0.03	0.63	0.75
Seeds/siliqua	IR	0.73	0.60	1.22	1.46	0.60	0.91	0.57
	RF	0.97	0.28	3.49	1.94	0.28	0.54	0.72
Days to 50% flowering	IR	8.98	15.29	0.59	17.96	15.29	1.30	0.48
	RF	5.08	12.25	0.41	10.15	12.25	1.55	0.40
1,000-seed weight	IR	0.05	0.19	0.26	0.10	0.19	1.95	0.32
	RF	0.03	0.30	0.11	0.07	0.30	2.96	0.17
Seed yield/plant	IR	10.58	85.62	0.12	21.16	85.62	2.84	0.19
	RF	7.27	82.16	0.09	14.54	82.16	3.36	0.15
Biological yield/plant	IR	1.88	1.92	0.98	3.76	1.92	1.01	0.16
	RF	147.00	1732.25	0.08	294.00	1732.25	3.43	0.14
Harvest index	IR	2.09	7.29	0.29	4.18	7.29	1.87	0.35
	RF	1.06	27.84	0.04	2.12	27.84	5.13	0.07
Seed yield/hectare	IR	18923.9	346993.1	0.05	37847.8	346993	4.28	0.10
	RF	47004.54	407072.46	0.12	94009	407072	2.94	0.18
Oil content	IR	0.90	3.74	0.24	1.80	3.74	2.04	0.29
	RF	1.58	1.94	0.81	3.16	1.94	1.11	0.54

*IR, irrigated; RF, rainfed; h^2^, narrow sense heritability.*

Narrow-sense heritability (h^2^) for different traits varied from 0.09 to 0.69 and 0.07 to 0.75 under irrigated and rainfed conditions, respectively (Table 3). The estimate of the degree of dominance varied from 0.74 to 4.40 and from 0.54 to 5.13 under irrigated and rainfed conditions respectively across the traits (Table 3). Low to moderate *h*^2^ were observed for all the traits under irrigated and rainfed conditions, with few exceptions. High average degree of dominance along with low *h*^2^ were observed for point to first siliqua on main shoot (cm), total number of siliquae per plant, seed yield per plant (g), biological yield per plant (g), and seed yield (kg/ha).

### General Combining Ability

The general combining ability analysis revealed that each CDL possessed significant GCA effects for one or more traits under both irrigated or rainfed conditions (Table 4). CDL89 and CDL141 were found to be a good general combiner for point to the first siliqua on the main shoot, harvest index, seed yield per plant, seed yield (kg/ha), and oil content under rainfed conditions. CDL161, CDL112, CDL186, CDL101, and CDL25 were also found to possess high GCA for seed yield (kg/ha) and some of its contributing traits under water deficit stress conditions (Table 4).

**TABLE 4:** General combining ability (GCA) effects of 15 parental lines and variance components for different traits in CDLs along with their hybrids under both rainfed and irrigated conditions.

Parents	Plant height (cm)	Point to first branch (cm)	Primary branches/plant	Secondary branches/plant	Main shoot length (cm)	Point to first siliqua on main shoot (cm)	Number of siliquae on main shoot	Total siliquae/plant	Siliqua length (cm)	Seeds/Siliqua	Day to 50% flowering	1,000-seed weight (g)	Seed yield/plant (g)	Biological yield/plant (g)	Harvest index	Seed yield (kg/ha)	Oil content (%)
CDL102	–14.2**	–14.63**	–0.13	2.9**	1.32	0.05	–4.69	71.12**	0.42**	2.12**	–4.3**	–0.25	–2.47	–19.53	0.63**	–222.3	–1.83
	(–12**)	(–13.2**)	(–0.87)	(–0.54)	(5.4**)	(–1.1**)	(–2.29)	(32.5**)	(0.41**)	(1.91**)	(–2.9**)	(–0.1)	(–3.04)	(5.69**)	(–2.8)	(–120.5)	(–1.8)
CDL161	–7.5**	–1.03	–0.56	–0.1	1.87*	–0.56**	0.45	–18.66	0.08	0.14	–4.12**	0.22**	3.38**	19.2**	–0.52	267.1**	–0.11
	(–3.3)	(–1.87**)	(0.27)	(0.97**)	(3.1**)	(–2.4**)	(3.67**)	(40.6**)	(–0.17)	(–0.28)	(–2.6**)	(0.43**)	(3.6**)	(21.5**)	(–0.19)	(72.5**)	(0.35)
CDL128	1.75	–1.25	–0.18	–0.48	–1.29	–0.86**	–0.43	8.36	0.05	–0.53	–2.2**	–0.26	3.5**	4.35*	1.16**	40.9	1.87**
	(1.5)	(–1.76**)	(–0.39)	(–0.05)	(–2.3)	(0.52)	(–5.83)	(–69.08)	(0.17**)	(0.54**)	(–2.4**)	(0.11)	(3.92**)	(1.7)	(2.1**)	(–142.7)	(2**)
CDL141	5.44	2.82	0.81**	–0.01	–4.68	–0.26**	0.22	–9.14	–0.2	–0.74	1.94	–0.02	0.99*	–1.77	0.47**	97.8**	1.51**
	(8.84)	(6.9)	(0.03)	(–0.89)	(–6.14)	(1.66)	(–2.97)	(–112.47)	(–0.15)	(–0.51)	(2.71)	(–0.31)	(–5.76)	(–29.34)	(–0.37)	(–265.8)	(0.5*)
CDL112	18.81	–0.6	0.39**	1.42**	3.31**	–1.33**	5.42**	51.38**	–0.35	–0.14	3.01	–0.14	4.76**	30.13**	–0.76	83.2**	1.5**
	(18.27)	(7.41)	(0.21)	(0.14)	(4.5**)	(–0.92**)	(3.27**)	(80.5**)	(–0.36)	(–0.37)	(4.73)	(–0.33)	(0.45)	(19.1**)	(–1.87)	(164**)	(–0.24)
CDL186	9.21	10.07	0.49**	–0.22	–1.25	0.23	2.33*	–32.96	–0.27	–0.13	2.07	0.23**	1.29**	2.26	0.77**	208.4**	–0.1
	(9.07)	(3.41)	(0.8**)	(1.31**)	(–3.78)	(–1.15**)	(–0.28)	(–18.8)	(0)	(0.51**)	(3.55)	(0.2**)	(–0.3)	(3.05)	(–0.97)	(54.9*)	(–0.5)
CDL182	4.58	8.6	0.34**	–0.61	–1.35	–0.01	–1.2	–71.05	0	0.30	1.25	0.07	–0.84	–7.1	0.64**	–24.18	–0.8
	(3.52)	(9.07)	(0.05)	(–1.37)	(–1.87)	(–1.35**)	(–1.78)	(–62.07)	(–0.11)	(0.45*)	(1.82)	(0.18**)	(3.04**)	(5.78**)	(1.1**)	(–66.9)	(–0.2)
CDL121	2.85	3.33	–0.12	–0.08	–0.6	0.29	–0.07	–27.22	–0.41	–1.87	1.25	0.16**	–1.92	–2.22	–0.61	–186	–0.88
	(5.61)	(9.56)	(–0.32)	(–0.73)	(1.26)	(0.94)	(1.85*)	(–49.1)	(–0.37)	(–1.77)	(2.57)	(0.2**)	(–0.71)	(–6.54)	(0.5**)	(–4.58)	(0.04)
CDL103	–6.02**	–1.03	–0.52	–1.19	0.54	0.35	–1.52	–35.57	0.16**	0.71**	–0.8**	0.05	–4.59	–14.92	–1.22	–524.9	–1.65
	(–7.2**)	(–5.11**)	(–0.35)	(–0.92)	(3.3**)	(2.16)	(–0.99)	(–48.38)	(0.12**)	(0.76**)	(–1.5**)	(0.15**)	(–1.71)	(–7.07)	(–0.12)	(–307.1)	(–0.4)
CDL101	0.12	4.81	0.53**	–0.13	–3.04	–0.11	1.75	–10.94	0.12**	0.22	1.25	–0.21	–1.04	–8.75	0.55**	164.9**	1.8**
	(–2.19)	(3.94)	(0.5**)	(–0.27)	(–2.97)	(–1.83**)	(5.58**)	(11.45*)	(0.07)	(–0.27)	(1.79)	(–0.13)	(1.4*)	(–4.6)	(1.7**)	(333**)	(1.2**)
CDL25	4.64	3.51	–0.2	0.43*	4.13**	1.07	5.65**	34.03**	–0.41	–0.75	0.74	–0.24	2.04**	3.22	1.59**	259.3**	0.4
	(4.09)	(–1.25**)	(–0.33)	(0.34)	(1.21)	(0.61)	(5.58**)	(86.6**)	(–0.22)	(–0.13)	(–0.22)	(–0.23)	(5.68**)	(15.1**)	(1.9**)	(313**)	(1.0**)
CDL89	–4.25*	–2.62**	0.15	0.07	–3.95	–0.79**	0.15	6.04	–0.12	–1.19	1.74	0.31**	0.91*	0.41	0.36*	221.4**	1.25**
	(–3.02)	(2.25)	(0.5**)	(0.38)	(–3.84)	(–0.85**)	(2.69**)	(–18.41)	(–0.12)	(–1.16)	(2.96)	(0.11*)	(–0.76)	(–12.6)	(1.4**)	(205**)	(0.6*)
CDL104	–6.5**	–2.04**	–0.22	–0.04	–1.48	–0.46**	–2.61	36.33**	0.11*	0.01	1.43	–0.07	–0.89	0.75	–0.61	40.56	–0.5
	(–8**)	(–5**)	(0.26)	(0.89**)	(–3.21)	(–1.46**)	(–3.06)	(43.8**)	(0.11**)	(–0.3)	(–1.9**)	(–0.22)	(–1.45)	(1.63)	(–1)	(35.9)	(–0.9)
CDL105	–9.02**	–6.56**	–0.57	–1.3	3.31**	0.94**	–2.69	–6.47	0.59**	1.56**	–1.61**	–0.06	–3.97	1.14	–2.47	–458.4	–0.96
	(–13**)	(–9.79**)	(–0.59)	(–1.11)	(1.18)	(0.74)	(–5.23)	(–14.96)	(0.49**)	(0.72**)	(–5.1**)	(–0.16)	(–5.46)	(–19.63)	(–1.2)	(–332.4)	(–0.79)
CDL106	0.13	–3.38**	–0.22	–0.66	3.18**	1.46	–2.75	4.74	0.21**	0.28	–1.61**	0.19**	–1.13	–7.17	0.02	32.1	–1.31
	(–2.19)	(–4.52**)	(0.18)	(1.84**)	(4.2**)	(0.6)	(–0.22)	(97.7**)	(0.11**)	(–0.09)	(–3.4**)	(0.09*)	(1.12*)	(6.29**)	(–0.23)	(60.9*)	(–0.91)
SE	1.98	0.64	0.08	0.17	0.72	0.07	0.82	3.97	0.04	0.16	0.42	0.05	0.42	1.65	0.14	27.01	0.20
	(1.6)	(0.32)	(0.13)	(0.18)	(0.83)	(0.12)	(0.84)	(5.14)	(0.03)	(0.17)	(0.48)	(0.04)	(0.48)	(1.84)	(0.16)	(26.72)	(0.21)
CD (P = 0.05)	4.24	1.36	0.16	0.36	1.55	0.16	1.75	8.51	0.08	0.35	0.91	0.11	0.89	3.55	0.29	53.2	0.43
	(3.44)	(0.69)	(0.28)	(0.38)	(1.78)	(0.26)	(1.8)	(11.02)	(0.07)	(0.36)	(1.03)	(0.08)	(1.03)	(3.94)	(0.33)	(52.63)	(0.45)
CD (P = 0.01)	5.88	1.89	0.23	0.50	2.15	0.22	2.43	11.81	0.11	0.49	1.26	0.15	1.23	4.92	0.40	70.13	0.60
	(4.77)	(0.96)	(0.39)	(0.53)	(2.48)	(0.36)	(2.49)	(15.3)	(0.10)	(0.50)	(1.44)	(0.11)	(1.43)	(5.46)	(0.46)	(69.38)	(0.63)

Values represent GCA effects under rainfed condition; Values in parenthesis represent GCA effects under irrigated condition; **Significant at P = 0.01 and *significant at P = 0.05.

Under irrigated condition, CDL161 and CDL106 expressed good general combining ability for point to the first branch, secondary branches/plant, main shoot length, total siliquae/plant, days to 50% flowering, 1,000-seed weight, seed yield/plant, biological yield/plant, and seed yield (kg/ha) (Table 4). CDL112, CDL186, CDL101, CDL25, and CDL89 also possessed higher GCA for seed yield (kg/ha) along with some of its contributing traits under water sufficient conditions (Table 4).

### Specific Combining Ability and Heterobeltiosis

Under the rainfed condition, a total of 36 hybrids exhibited significant SCA effects and high heterobeltiosis (>15%) for seed yield (kg/ha). Highest heterobeltiosis (99.55%) was observed for the cross CDL106 × CDL104 under limited water conditions. Out of 36, nine hybrids, *viz*., CDL102 × CDL89, CDL102 × CDL106, CDL112 × CDL89, CDL186 × CDL121, CDL182 × CDL121, CDL 121 × CDL 103, CDL25 × CDL104, CDL89 × CDL104, and CDL104 × CDL106 exhibited highly significant SCA effects and more than 50% heterobeltiosis (Table 5). Similarly, under irrigated condition, 30 hybrids expressed significant SCA effects and heterobeltiosis > 15%. Highest heterobeltiosis (82.34%) for seed yield (kg/ha) was observed for the hybrid derived from parents CDL141 and CDL161 under favourable environment. Six hybrids, *viz*., CDL161 × CDL186, CDL161 × CDL141, CDL141 × CDL182, CDL182 × CDL103, CDL182 × CDL106, and CDL104 × CDL106 recorded highly significant SCA effects and >50% heterobeltiosis for seed yield (kg/ha) under irrigated condition. It is also important to note that hybrid derived from cross CDL104 × CDL106 observed highly significant SCA effects and >50% heterobeltiosis under both rainfed and irrigated conditions (Table 5).

**TABLE 5:** Estimates of heterobeltiosis and specific combining ability (SCA) effects of 105 hybrids of CDLs for seed yield (kg/ha) under irrigated and rainfed conditions.

	CDL102	CDL161	CDL128	CDL141	CDL112	CDL186	CDL182	CDL121	CDL103	CDL101	CDL25	CDL89	CDL104	CDL105	CDL106
CDL102		–3.36	–27.74	–4.26	–13.98	42.82**	–36.94	–40.92	–8.82	20.32**	46.03**	51.62**	–15.33	–19.52	55.86**
		(–297.5)	(–532.5)	(–322.8)	(–235.9)	(605.6**)	(–970.1)	(–1000)	(105.6)	(626.9**)	(743.6**)	(988.7**)	(–615.4)	(–216.5)	(1093**)
CDL161	45**		34.03**	43.07**	–9.87	39.72**	12.32*	8.98	12.95*	40.04**	–3.36	29.75**	–0.35	22.99**	14.67*
	(685**)		(778.2**)	(615.7**)	(–608.6)	(416.3**)	(–78.9)	(–6)	(438.5**)	(693**)	(–779.1)	(138.3)	(–480.4)	(638.6**)	(–73)
CDL128	17.23*	44.04**		15.69**	–38.8	20.11**	32.6**	–31.93	–2.7	6.86	12.01*	–2.8	10.96*	–28.98	–27.36
	(289**)	(712.8**)		(412.9**)	(–1160.2)	(431.3**)	(1027**)	(–690.7)	(500**)	(88.7)	(144.3)	(–249.5)	(332.5**)	(–332.4)	(–775.7)
CDL141	0.5	82.34**	31.22**		19.75**	24.89**	30.16**	21.08**	12.6*	–8.68	40.25**	7.52	13.01*	–6.99	14.13*
	(8.2)	(788**)	(756**)		(402.3**)	(–31.2)	(415.3**)	(268.8**)	(398**)	(–510)	(298**)	(–438.7)	(–157.8)	(–154.4)	(–121.6)
CDL112	20.9**	0.62	–22.62	–0.86		1.76	7.33	–0.1	–2.05	–9.97	7.41	52.49**	8.5	–4.11	17.3**
	(565**)	(–178)	(–593.3)	(120.1)		(–219.3)	(171.6)	(122.3)	(406**)	(–509.2)	(–109.7)	(1209**)	(140.2)	(280.8**)	(398.7**)
CDL186	31.68**	53.41**	23.27**	17.23**	–15.56		–26.45	67.19**	–11.02	22.39**	29.24**	23.76**	49.03**	27.94**	57.43**
	(399**)	(481.7**)	(252.5*)	(46.5)	(–599.5)		(–1134.2)	(994.4**)	(–461.2)	(254.5*)	(–101)	(–141.4)	(351.1**)	(366.2**)	(552.4**)
CDL182	–25.76	27.3**	26.66**	55.83**	10.44	8.19		69.03**	–6.12	12.03*	18.91**	33.01**	–10.38	–31.04	4.92
	(–789.5)	(–396.4)	(452.3**)	(375**)	(227.9*)	(–345.7)		(1687**)	(115.9)	(195.4)	(–32.3)	(363.9**)	(–558)	(–584)	(–160.6)
CDL121	–49.24	5.92	–8.25	–12.29	22.94**	49.45**	48.49**		66.24**	–28.21	45.92**	5.86	31.69**	22.45**	69.56**
	(–1282.4)	(–103.2)	(–240.5)	(–217.9)	(504.5**)	(997.5**)	(1095**)		(394.4**)	(–776.1)	(705**)	(–196.4)	(–76.7)	(294.5**)	(163.5)
CDL103	–8.83	31.02**	–12.24	35.44**	–14.75	–17.28	60.94**	21.44**		–34.08	13.37*	–28.3	–42.11	36.64**	48.02**
	(–163.2)	(–89.5)	(–202.1)	(237.5*)	(–215.2)	(–649.9)	(505.3**)	(662.5**)		(–602.7)	(241.3*)	(–715.3)	(–1193.3)	(919.5**)	(142.9)
CDL101	1.11	25.21**	–5.43	0.8	5.82	42.82**	4.42	–5.83	18.1**		7.79	12.49*	42.31**	–14.79	28.11**
	(–89.5)	(383.6**)	(–247.8)	(47.3)	(–244.2)	(887.7**)	(–51.6)	(–397.1)	(566.6**)		(–207.5)	(–37.4)	(983.5**)	(–125.9)	(591.9**)
CDL25	37.81**	15.18**	–5.72	0.59	6.94	–6.95	–15.19	11.41	11.21	38**		27.91**	71.06**	–1.75	27.44**
	(995**)	(168.5)	(–201.4)	(98.3)	(–154.4)	(–433.6)	(–542.3)	(140)	(437**)	(546.7**)		(–88.1)	(1098**)	(–198.1)	(30.9)
CDL89	4.09	–12.61	–34.65	–43.03	31.22**	8.61	–12.29	–22.27	–13.66	–23.92	–3.97		58.19**	–16.7	33.08**
	(569**)	(–157.7)	(–646.3)	(–790.7)	(1150**)	(537.5**)	(–7.9)	(–388.9)	(188.6)	(–779.3)	(–121.8)		(891**)	(–490.7)	(269**)
CDL104	–31.15	–11.23	–1.19	–22.57	–15.56	–2.78	27.42**	8.63	–25.59	17.69**	16.57**	5.46		23.97**	99.55**
	(–774.8)	(–443.8)	(35.4)	(–404)	(–580.5)	(–204.2)	(712.1**)	(155.5)	(–441.9)	(212.3*)	(243.9*)	(455.9**)		(98.5)	(1043**)
CDL105	–45.27	–3.1	–21.64	–24.97	2.56	–19.94	–29.8	–22.11	–29.63	–27.71	–10.82	–27.14	–35.78		–17.25
	(–615.7)	(425.6**)	(98.2)	(123.8)	(499**)	(–49.7)	(–216.5)	(–53.9)	(28.7)	(–555.4)	(–40.5)	(–216.7)	(–494.4)		(–724.3)
CDL106	36.47**	26.96**	–0.28	3.82	9.42	28.72**	59.53**	–11.5	25.87**	42.33**	3.67	–8.52	71.83**	–44.51	
	(502**)	(–227.1)	(–295.1)	(–358.4)	(72.3)	(–34.7)	(573.4**)	(–525)	(130.3)	(867.9**)	(–142.2)	(–15.6)	(1752**)	(–775)	

*Heterobeltiosis and SCA effects for rainfed (above diagonal) and irrigated condition (below diagonal) Values in parenthesis represent SCA effects.*

**Significant at p = 0.01 and *significant p = 0.01 and *significant at P = 0.05.

High heterobeltiosis (>15%) and higher SCA effects for seed yield were reported for hybrids derived from different combinations of parental CDLs, irrespective of their GCA effect levels (Table 6). Furthermore, 14 hybrids expressed significant SCA effects and high heterobeltiosis (>15%) under both rainfed and irrigated conditions (Table 6).

**TABLE 6:** Combining ability and heterobeltiosis of hybrids derived from CDLs with different levels of GCA effects for seed yield (kg/ha) under irrigated and rainfed conditions.

Environment	GCA effects of CDLs for Seed yield (kg/ha)		Hybrids with highly significant SCA effects and > 15% heterobeltiosis		
		
	Significant	Non-significant	Hybrids developed from crossing CDLs with significant GCA effects	Hybrids developed from crossing CDLs with significant and non-significant GCA effects	Hybrids developed from crossing CDLs with non-significant GCA effects
Rainfed condition (RF)	CDL161, CDL141, CDL112, CDL186, CDL101, CDL25 and CDL89	CDL102, CDL128, CDL182, CDL121, CDL103, CDL104, CDL105 and CDL106	CDL161 × CDL141, CDL161 × CDL186, CDL161 × CDL101, CDL141 × CDL112, CDL141 × CDL25, CDL112 × CDL89, CDL186 × CDL101 (7 Hybrids)	CDL161 × CDL128, CDL161 × CDL105, CDL141 × CDL128, CDL141 × CDL182, CDL141 × CDL121, CDL112 × CDL106, CDL186 × CDL102, CDL186 × CDL128, CDL186 × CDL121, CDL186 × CDL104, CDL186 × CDL105, CDL186 × CDL106, CDL101 × CDL102, CDL101 × CDL104, CDL101 × CDL106, CDL25 × CDL102, CDL25 × CDL121, CDL25 × CDL104, CDL89 × CDL102, CDL89 × CDL182, CDL89 × CDL104, CDL89 × CDL106 (22 Hybrids)	CDL102 × CDL106, CDL128 × CDL182, CDL182 × CDL121, CDL121 × CDL103, CDL121 × CDL105, CDL103 × CDL105, CDL104 × CDL106 (7 Hybrids)
Irrigated condition (IR)	CDL161, CDL112, CDL186, CDL101, CDL25, CDL89, and CDL106	CDL102, CDL128, CDL141, CDL182, CDL121, CDL103, CDL104, and CDL105	CDL161 × CDL101, CDL161 × CDL186, CDL112 × CDL89, CDL101 × CDL25, CDL186 × CDL101, CDL101 × CDL106 (6 Hybrids)	CDL161 × CDL102, CDL161 × CDL128, CDL161 × CDL141 CDL112 × CDL102, CDL112 × CDL121, CDL101 × CDL103, CDL101 × CDL104, CDL25 × CDL102, CDL25 × CDL104, CDL186 × CDL102, CDL186 × CDL128, CDL186 × CDL121, CDL106 × CDL102, CDL106 × CDL182, CDL106 × CDL104 (15 Hybrids)	CDL102 × CDL128, CDL128 × CDL141, CDL128 × CDL182, CDL141 × CDL182, CDL141 × CDL103, CDL182 × CDL121, CDL182 × CDL103, CDL182 × CDL104, CDL121 × CDL103 (9 Hybrids)
Number common in RF and IR conditions	6 CDLs	7 CDLs	4 hybrids	7 hybrids	3 hybrids

### Mean Performance

The water requirement of rapeseed mustard is about 190–400 mm (Shekhawat et al., 2012; Sharma et al., 2018) and it is sensitive to water deficit stress at critical stages like pre-flowering and siliquae formation stages. In the presented experiment, the rainfed plots received 112.4 mm of water through rains up to physiological maturity stage, whereas two additional irrigations of 50 mm each were applied in the irrigated plots. Thus, the irrigated plots received a total of 212.4 mm of water throughout the crop developmental stages and met optimum water requirement. The data recorded on 17 morpho-physiological traits under rainfed and irrigated conditions were averaged separately for 15 parents and 105 hybrids. The results suggested that parental CDLs exhibited a higher mean value under irrigated than in rainfed condition for traits like plant height (cm), number of primary branches, number of secondary branches, main shoot length (cm), point to first siliqua on the main shoot (cm), total siliquae per plant, days to 50% flowering, and seed yield (kg/ha). Whereas, under the rainfed condition, the mean value of CDLs was higher for point to first branch (cm), siliquae on the main shoot, siliqua length (cm), seeds/siliqua, 1,000-seed weight (g), seed yield/plant (g), biological yield/plant (g), harvest index, and oil content traits (Table 7) than in irrigated condition. Under irrigated condition, the overall mean of the hybrids was higher for plant height, primary branches per plant, secondary branches per plant, main shoot length, point to first siliqua on main shoot, total siliquae per plant and days to 50% flowering than in rainfed condition. Whereas traits like point to first branch (cm), number of siliquae on the main shoot, siliqua length (cm), seeds per siliqua, 1,000-seed weight (g), seed yield per plant (g), biological yield per plant (g), harvest index, seed yield (kg/ha), and oil content (%) observed higher mean values for hybrids when evaluated under rainfed condition (Table 7). Overall performance of CDLs and hybrids was found better when evaluated under rainfed condition for important yield and yield attributing traits including plant height (cm), total siliquae on main shoot (no.), siliqua length (cm), seeds/siliqua, days to 50% flowering, 1,000-seed weight (g), seed yield/plant, biological yield/plant (g), harvest index, and oil content (%) traits (Table 7).

**TABLE 7:** Mean values recorded on 15 parents and 105 hybrids for different traits under irrigated and rainfed conditions.

Traits	Parents		Hybrids	
	IR	RF	IR	RF
Plant height (cm)	217.91 +	216.87	231.81*	229.88
Point to first branch (cm)	34.08	52.61 +	32.09	47.86*
Primary branches/plant	7.02 +	6.49	7.68*	6.96
Secondary branches/plant	15.07 +	11.46	18.29*	12.19
Main shoot length (cm)	75.86 +	75.76	80.28*	78.46
Point to first siliqua on main shoot (cm)	8.56 +	7.51	8.34*	7.35
Number of siliqua on main shoot	48.65	49.97 +	54.30	55.54*
Total siliqua/plant	457.48 +	456.80	539.24*	472.25
Siliqua length (cm)	4.03	4.19 +	4.17	4.35*
Seeds/siliqua	15.12	15.98 +	16.00	16.08*
Days to 50% flowering	52.67 +	53.96	57.31*	55.85
1,000-seed weight (g)	3.70	4.49 +	4.00	4.38*
Seed yield/plant (g)	20.36	25.79 +	30.17	32.14*
Biological yield/plant (g)	118.12	144.27 +	156.56	166.08*
Harvest index	17.38	18.14 +	19.23	19.77*
Seed yield (kg/ha)	2338+	2300	2718	2894*
Oil content (%)	36.63	38.75 +	38.56	39.07*

*Traits for which higher mean value observed in hybrids.

*^+^Traits for which higher mean value observed in parental CDLs.*

*RF, rainfed; IR, irrigated.*

### Tolerance Indices and Water Use Efficiency

Drought tolerance indices, *viz*., drought susceptibility index (DSI), drought tolerance index (DTI), tolerance index (TOL), and mean relative performance (MRP) for seed yield (kg/ha) were estimated. Correlation coefficients between seed yield under irrigated condition (Y_*IRR*_), seed yield under rainfed condition (Y_*RF*_), WUE_*RF*_, WUE_*IRR*_, DSI, DTI, TOL, and MRP were calculated. Yield under rainfed condition (Y_*RF*_) were showing significant and positive association with WUE_*RF*_, WUE_*IR*_, DTI, MRP, and TOL. Similarly, significantly positive association of Y_*IR*_ with DTI and MRP was observed (Supplementary Table 2). WUE_*RF*_ exhibited a positive and significant correlation with DSI, DTI, TOL, and MRP, while WUE_*IR*_ showed a positive and significant correlation with DTI and MRP. DTI and MRP were identified to be significantly associated with all the traits except DSI, thus, indicating the usefulness of these two indices in indirect selection for drought tolerance.

The WUE (kg m^–3^) was calculated for all hybrids along with parental CDLs both under rainfed and irrigated conditions. WUE among the parental lines raised under rainfed condition varied from 1.27 to 2.59 kg m^–3^ and recorded the highest and lowest values for CDL128 and CDL103, respectively (Table 8). Under irrigated condition it ranged from 0.73 to 1.50 kg m^–3^ with the highest and lowest values for CDL89 and CDL103, respectively. Among 105 hybrids evaluated under rainfed condition, WUE varied from 1.02 to 3.86 kg m^–3^ and the highest value was recorded for the cross CDL112 × CDL89. On the other hand, WUE varied from 0.59 to 2.13 kg m^–3^ under the irrigated condition and exhibited the highest value for CDL104 × CDL106 cross (Table 8). In general, the hybrids observed higher WUE than their parental CDLs. Overall, the mean of seed yield (kg/ha) and WUE of parental CDLs and hybrids was recorded higher under rainfed condition than in irrigated condition (Figure 1).

**TABLE 8:** Water use efficiency, drought tolerance indices, and seed yield performance of CDLs and their *F*_1_ hybrids under rainfed (RF) and irrigated (IR) conditions.

CDLs/Cross	Seed yield (kg/ha)		WUE (kg/m^3^)		DTI	MRP	CDLs/Cross	Seed yield (kg/ha)		WUE (kg/m^3^)		DTI	MRP
	RF	IR	RF	IR				RF	IR	RF	IR		
CDL102	2388	2281	2.12	1.07	0.76	1.70	CDL161 × CDL186	3711	3280	3.30	1.54	1.71	2.54
CDL161	2656	1791	2.36	0.84	0.67	1.61	CDL161 × CDL182	2983	2280	2.65	1.07	0.95	1.91
CDL128	2914	2300	2.59	1.08	0.94	1.89	CDL161 × CDL121	2894	2635	2.58	1.24	1.07	2.01
CDL141	2478	1724	2.20	0.81	0.60	1.52	CDL161 × CDL103	3000	2346	2.67	1.10	0.99	1.94
CDL112	2842	2713	2.53	1.28	1.08	2.02	CDL161 × CDL101	3944	3460	3.51	1.63	1.91	2.69
CDL186	2294	2138	2.04	1.01	0.69	1.61	CDL161 × CDL25	2567	3224	2.28	1.52	1.16	2.12
CDL182	2542	1741	2.26	0.82	0.62	1.55	CDL161 × CDL89	3446	2791	3.07	1.31	1.35	2.27
CDL121	1506	2488	1.34	1.17	0.53	1.47	CDL161 × CDL104	2647	2335	2.35	1.10	0.87	1.81
CDL103	1425	1559	1.27	0.73	0.31	1.09	CDL161 × CDL105	3267	2836	2.91	1.34	1.30	2.22
CDL101	2817	2763	2.51	1.30	1.09	2.03	CDL161 × CDL106	3046	2577	2.71	1.21	1.10	2.05
CDL25	2465	2799	2.19	1.32	0.97	1.92	CDL128 × CDL141	3371	3018	3.00	1.42	1.43	2.33
CDL89	2511	3194	2.23	1.50	1.12	2.09	CDL128 × CDL112	1783	2099	1.59	0.99	0.52	1.42
CDL104	1972	2630	1.75	1.24	0.73	1.68	CDL128 × CDL186	3500	2835	3.11	1.33	1.39	2.30
CDL105	2017	2927	1.79	1.38	0.83	1.81	CDL128 × CDL182	3864	2913	3.44	1.37	1.58	2.46
CDL106	1668	2030	1.48	0.96	0.47	1.35	CDL128 × CDL121	1984	2283	1.76	1.07	0.63	1.56
CDL102 × CDL161	2567	3307	2.28	1.56	1.19	2.15	CDL128 × CDL103	2835	2019	2.52	0.95	0.80	1.76
CDL102 × CDL128	2106	2696	1.87	1.27	0.80	1.76	CDL128 × CDL101	3114	2613	2.77	1.23	1.14	2.08
CDL102 × CDL141	2372	2292	2.11	1.08	0.76	1.70	CDL128 × CDL25	3264	2639	2.90	1.24	1.21	2.15
CDL102 × CDL112	2444	3280	2.17	1.54	1.12	2.10	CDL128 × CDL89	2832	2087	2.52	0.98	0.83	1.79
CDL102 × CDL186	3411	3004	3.03	1.41	1.44	2.33	CDL128 × CDL104	3233	2599	2.88	1.22	1.18	2.12
CDL102 × CDL182	1603	1694	1.43	0.80	0.38	1.20	CDL128 × CDL105	2069	2294	1.84	1.08	0.67	1.59
CDL102 × CDL121	1411	1263	1.26	0.59	0.25	0.97	CDL128 × CDL106	2117	2294	1.88	1.08	0.68	1.61
CDL102 × CDL103	2178	2080	1.94	0.98	0.64	1.55	CDL141 × CDL112	3403	2689	3.03	1.27	1.28	2.21
CDL102 × CDL101	3389	2794	3.02	1.32	1.33	2.25	CDL141 × CDL186	3094	2506	2.75	1.18	1.09	2.04
CDL102 × CDL25	3600	3857	3.20	1.82	1.95	2.72	CDL141 × CDL182	3308	2713	2.94	1.28	1.26	2.19
CDL102 × CDL89	3807	3324	3.39	1.57	1.77	2.60	CDL141 × CDL121	3000	2182	2.67	1.03	0.92	1.88
CDL102 × CDL104	2022	1811	1.80	0.85	0.51	1.40	CDL141 × CDL103	2790	2335	2.48	1.10	0.91	1.86
CDL102 × CDL105	1922	1602	1.71	0.75	0.43	1.28	CDL141 × CDL101	2572	2785	2.29	1.31	1.00	1.96
CDL102 × CDL106	3722	3113	3.31	1.47	1.62	2.49	CDL141 × CDL25	3475	2816	3.09	1.33	1.37	2.29
CDL161 × CDL128	3906	3313	3.47	1.56	1.81	2.63	CDL141 × CDL89	2700	1819	2.40	0.86	0.69	1.64
CDL161 × CDL141	3800	3265	3.38	1.54	1.74	2.57	CDL141 × CDL104	2800	2037	2.49	0.96	0.80	1.76
CDL161 × CDL112	2561	2730	2.28	1.29	0.98	1.93	CDL141 × CDL105	2304	2196	2.05	1.03	0.71	1.64
CDL141 × CDL106	2828	2107	2.52	0.99	0.84	1.79	CDL121 × CDL103	2503	3021	2.23	1.42	1.06	2.02
CDL112 × CDL186	2892	2291	2.57	1.08	0.93	1.88	CDL121 × CDL101	2022	2602	1.80	1.22	0.74	1.69
CDL112 × CDL182	3050	2996	2.71	1.41	1.28	2.20	CDL121 × CDL25	3597	3119	3.20	1.47	1.57	2.44
CDL112 × CDL121	2839	3335	2.53	1.57	1.33	2.26	CDL121 × CDL89	2658	2482	2.37	1.17	0.93	1.87
CDL112 × CDL103	2783	2313	2.48	1.09	0.90	1.85	CDL121 × CDL104	2597	2857	2.31	1.35	1.04	1.99
CDL112 × CDL101	2558	2924	2.28	1.38	1.05	2.00	CDL121 × CDL105	2469	2280	2.20	1.07	0.79	1.73
CDL112 × CDL25	3052	2993	2.72	1.41	1.28	2.20	CDL121 × CDL106	2829	2202	2.52	1.04	0.87	1.83
CDL112 × CDL89	4333	4191	3.86	1.97	2.55	3.11	CDL103 × CDL101	1857	3263	1.65	1.54	0.85	1.88
CDL112 × CDL104	3083	2291	2.74	1.08	0.99	1.95	CDL103 × CDL25	2795	3113	2.49	1.47	1.22	2.16
CDL112 × CDL105	2725	3002	2.42	1.41	1.15	2.09	CDL103 × CDL89	1801	2757	1.60	1.30	0.70	1.67
CDL112 × CDL106	3333	2968	2.97	1.40	1.39	2.29	CDL103 × CDL104	1142	1957	1.02	0.92	0.31	1.14
CDL186 × CDL182	1869	2313	1.66	1.09	0.61	1.53	CDL103 × CDL105	2756	2060	2.45	0.97	0.80	1.75
CDL186 × CDL121	3836	3718	3.41	1.75	2.00	2.75	CDL103 × CDL106	2469	2555	2.20	1.20	0.88	1.83
CDL186 × CDL103	2042	1768	1.82	0.83	0.51	1.39	CDL101 × CDL25	3036	3863	2.70	1.82	1.64	2.52
CDL186 × CDL101	3447	3946	3.07	1.86	1.91	2.70	CDL101 × CDL89	3168	2430	2.82	1.14	1.08	2.03
CDL186 × CDL25	3186	2604	2.83	1.23	1.16	2.11	CDL101 × CDL104	4008	3252	3.57	1.53	1.83	2.64
CDL186 × CDL89	3108	3468	2.76	1.63	1.51	2.40	CDL101 × CDL105	2400	2116	2.14	1.00	0.71	1.64
CDL186 × CDL104	3419	2557	3.04	1.20	1.23	2.17	CDL101 × CDL106	3608	3932	3.21	1.85	1.99	2.75
CDL186 × CDL105	2936	2343	2.61	1.10	0.96	1.92	CDL25 × CDL89	3212	3067	2.86	1.44	1.38	2.29
CDL186 × CDL106	3612	2752	3.21	1.30	1.39	2.31	CDL25 × CDL104	4217	3263	3.75	1.54	1.93	2.72
CDL182 × CDL121	4296	3694	3.82	1.74	2.23	2.91	CDL25 × CDL105	2422	2610	2.16	1.23	0.89	1.84
CDL182 × CDL103	2386	2802	2.12	1.32	0.94	1.90	CDL25 × CDL106	3142	2902	2.80	1.37	1.28	2.20
CDL182 × CDL101	3156	2885	2.81	1.36	1.28	2.20	CDL89 × CDL104	3972	3368	3.53	1.59	1.88	2.67
CDL182 × CDL25	3022	2374	2.69	1.12	1.01	1.96	CDL89 × CDL105	2092	2327	1.86	1.10	0.68	1.61
CDL182 × CDL89	3381	2801	3.01	1.32	1.33	2.25	CDL89 × CDL106	3342	2921	2.97	1.38	1.37	2.28
CDL182 × CDL104	2278	3352	2.03	1.58	1.07	2.06	CDL104 × CDL105	2500	1880	2.22	0.88	0.66	1.59
CDL182 × CDL105	1753	2055	1.56	0.97	0.50	1.39	CDL104 × CDL106	3936	4520	3.50	2.13	2.49	3.09
CDL182 × CDL106	2667	3238	2.37	1.52	1.21	2.16	CDL105 × CDL106	1669	1624	1.48	0.76	0.38	1.20
							Overall mean	2819	2670	2.51	1.26	1.09	2.00
							CD	321	318				

*WUE, water use efficiency; DTI, drought tolerance index; MRP, mean relative performance.*

Further, drought tolerance index (DTI) and mean relative performance (MRP) were calculated from average values of individual parent and hybrid for all the 17 traits. All the traits, except point to first branch and point to first siliqua on the main shoot, exhibited higher DTI and MRP for hybrids than their corresponding parents (Figure 2). DTI for seed yield (kg/ha) varied from 0.25 to 2.55 in hybrids, whereas it varied from 0.31 to 1.12 in CDLs (Table 8). Some of the hybrids such as CDL112 × CDL89 (DTI: 2.55), CDL104 × CDL106 (DTI: 2.49) CDL182 × CDL121 (DTI: 2.23), CDL186 × CDL121 (DTI: 2.0), CDL101 × CDL106 (DTI: 1.99), CDL102 × CDL25 (DTI: 1.95), CDL25 × CDL104 (DTI: 1.93), CDL161 × CDL101 (DTI: 1.91), and CDL186 × CDL101 (DTI: 1.91) has a DTI value of >1, indicating their superiority under both moisture-stress and non-stress conditions. Among the CDLs highest MRP was recorded for CDL89 (2.09) and the lowest was observed for CDL103 (1.09). Among the hybrids, CDL112 × CDL89 recorded with the highest (3.11) MRP (Table 8). Further, nineteen hybrids, viz., CDL102 × CDL186, CDL102 × CDL25, CDL102 × CDL89, CDL102 × CDL106, CDL161 × CDL128, CDL161 × CDL141, CDL161 × CDL186, CDL161 × CDL101, CDL128 × CDL141, CDL186 × CDL121, CDL186 × CDL101, CDL182 × CDL121, CDL121 × CDL25, CDL101 × CDL104, CDL101 × CDL106, CDL25 × CDL89, CDL25 × CDL104, CDL89 × CDL104, and CDL104 × CDL106 were identified to be significantly better in productivity and possessing higher values of DTI and MRP (Table 8).

## Discussion

The present study revealed the usefulness of CDLs for improving heterosis and WUE in *B. juncea*. The existence of significant differences (*p* < 0.001) among parental CDLs and hybrids for all the 17 traits studied under irrigated and rainfed conditions indicated wide variability for the test traits in parents and their hybrids. The significance of mean squares among parents and hybrids suggested the suitability of the CDLs for combining ability and other analyses. Under both water regimes, the mean squares of parents vs. hybrids were found significant for several traits, revealed good scope for the manifestation of heterosis in hybrids generated from *B. carinata*-derived *B. juncea* lines. Further, under both irrigated and rainfed (Tables 1, 2) conditions, the mean sum of squares due to hybrids was significant for all the traits, indicating the importance of both additive and non-additive variance. Earlier studies have also reported similar trends for additive and non-additive variance for the traits under study (Rao and Gulati, 2001; Yadav et al., 2005; Singh et al., 2006, 2013; Singh N. et al., 2015; Verma et al., 2011). Polygenic inheritance of drought tolerance is reported in many crop species including mustard (Fletcher et al., 2015). The quantitative traits are complex to understand hence methods like quantitative trait loci (QTL) mapping, marker-assisted breeding, and introgression from wild or related species are being used to improve drought tolerance (Mwadzingeni et al., 2016).

### Combining Ability and Gene Action

Combining ability analysis partitions the genotypic variability into GCA (σ^2^gca) and SCA (σ^2^sca) variances representing additive and dominance effects, respectively. The higher value of σ^2^sca than σ^2^gca, along with an average degree of dominance more than unity observed for almost all the traits studied under irrigated and rainfed conditions, demonstrates the predominance of non-additive gene effects in the manifestation of heterosis (Table 3). For siliqua length and seeds per siliqua evaluated under both irrigated and rainfed condition, σ^2^gca were higher than σ^2^sca and average degree of dominance were less than unity demonstrating the role of additive gene effects in governing them. Similar results were also reported by Yadava et al. (2012) and Singh N. et al. (2015) in Indian mustard. Since additive and non-additive gene action were governing the yield contributing traits under irrigated and rainfed conditions, multiple or reciprocal recurrent selection could be useful for the improvement of these traits. The narrow-sense heritability (*h*^2^) found low to moderate for almost all the traits under both the water regimes, suggesting the importance of interaction effects (Sayar et al., 2007). Since the expected genetic gain per cycle of selection will be less important, the development of hybrids involving CDLs carrying *B. carinata* genomic segments holds promise for exploitation of heterosis under rainfed conditions.

Significant GCA effects were observed for CDL161, CDL141, CDL112, CDL186, CDL101, CDL25, and CDL89 for seed yield (kg/ha) along with some of its contributing traits under rainfed condition, whereas under irrigated condition, CDL161, CDL112, CDL186, CDL101, CDL25, CDL89, and CDL106 expressed good general combining ability for seed yield (kg/ha) and its contributing traits (Table 4). The results suggested the importance of additive or additive × additive gene effects which are governing these traits. Similar results have also been reported earlier using a different set of Indian mustard genotypes (Mahak et al., 2008; Singh et al., 2010, 2013; Yadava et al., 2012; Singh N. et al., 2015). Furthermore, the good general combiners for seed yield traits were not showing significant GCA effects for some of its contributing traits, hence, there is a scope of improving the combining ability among parents for yield contributing traits under both irrigated and rainfed conditions. The findings of the present study suggest that CDLs, *viz*., CDL161, CDL112, CDL186, CDL101, CDL25, and CDL89 possessing variable drought tolerance and expressing high GCA for seed yield along with other component traits under both rainfed and irrigated conditions can be incorporated in the breeding programme for accumulating desirable alleles for developing improved mustard cultivars suitable to water-scarce regions.

In the present study, significant SCA effects and high heterobeltiosis (>15%) were exhibited by 36 and 30 hybrids under rainfed and irrigated condition, respectively. Out of these, nine and six hybrids were expressing more than 50% heterobeltiosis when evaluated under rainfed and irrigated conditions, respectively (Table 5). This suggests that CDLs taken in this study can be exploited for developing hybrids for water-scarce regions. It is also important to note that 14 hybrids exhibited higher SCA effects and heterobeltiosis (>15%) under both water regimes (Table 6). These hybrids, expected to carry different introgression segments from *B. carinata*, have better resilience to changing water regimes and, thus, expressed better stability of performance. Earlier studies have recorded similar observations with a different set of conventional *B. juncea* genotypes (Singh et al., 2006; Srivastava et al., 2009). Teklewold and Becker (2005) reported significant SCA effects along with high heterobeltiosis for seed yield in *B. carinata* inbred lines, indicating substantial potential of Ethiopian mustard genotypes and their significance in the exploitation of hybrid vigour in *B. juncea* breeding programmes. Considerable heterosis was also reported in inter-subgenomic hybrids between *B. carinata*-derived introgression lines and *B. juncea* accessions (Wei et al., 2016). Therefore, the development of hybrids using *B. carnata* derived lines will be an ideal approach in realizing higher yields under drought conditions.

The higher SCA effects and heterobeltiosis (>15%) for seed yield were exhibited by hybrids derived from CDLs, irrespective of their GCA effect values. Thus, hybrids generated from CDLs with significant × significant, significant × non-significant, and non-significant × non-significant GCA effects expressed high SCA effects and heterobeltiosis (Table 6). It demonstrates that joint effects of native and alien heterotic loci are responsible for the expression of such SCA effects and heterobeltiosis in hybrids derived from CDLs. Langham (1961) and Lal et al. (2006) have explained this anomaly where parents with other than high × high GCA effects also resulted in better heterosis and concluded that cross between parents with significantly high × low trait value resulted in superior transgressive segregants and, thus, the genes governing high trait values may express under homozygous or heterozygous conditions. Further, the genes governing lower trait values can also be expressed to their full potential in the homozygous state, but in heterozygote state, they can express very high performance due to complementation of alleles or overdominance. The existence of heterotic loci in these *B. carinata* derived lines can thus be exploited in the *B. juncea* breeding program for improving the level of heterosis and its commercial use.

### Mean Performance, Tolerance Indices, and Water Use Efficiency

*Brassica carinata*, in general, adapted to tolerate drought conditions (Thakur et al., 2021), performed better under less availability of water and lodges under irrigated conditions due to its poor ability to withstand accumulated higher biomass. It has also been observed that irrigations in *B. carinata* genotypes also exhibits heavy regeneration, resulting in reduced plant height, poor seed set, and reduced seed size, which ultimately leads to reduction in seed yield per unit area. Conversely, bolder seed size is realised under rainfed than in irrigated conditions. In the present study, some of the *B. carinata* derived lines, along with their hybrids, observed reduced performance for seed yield under irrigated than in rainfed conditions. This could be attributed to the introduction of genomic segment(s) or substituted chromosomes from the C genome of *B. carinata* to *B. juncea*. Although, the possibility of later is remote when the biparental mating approach is being used for the development of such CDLs.

Parental CDLs and their hybrids exhibited higher mean values for seed yield (kg/ha) and other yield attributes under rainfed than in irrigated condition (Table 7). It is most probably due to presence of different introgression segments in CDLs which alter their performance in water deficit and surplus condition, whereas complementation of distinct genomic segments and expressed overdominance leads to heterosis in hybrids. Therefore, comparatively better performance of these hybrids under rainfed compared to irrigated conditions suggested that *B. carinata*-derived lines hold promise for the development of hybrids or cultivars for water-scarce regions.

The present study revealed that yield under rainfed condition (Y_*RF*_) was showing positive correlation with WUE_*RF*_ (*r* = 0.99), WUE_*IR*_ (*r* = 0.65), and stress tolerance indices, *viz*., DTI (*r* = 0.90), MRP (*r* = 0.92), and TOL (*r* = 0.54). Similarly, yield under irrigated conditions (Y_*IR*_) was positively correlated with DTI (*r* = 0.89) and MRP (*r* = 0.90). In addition, DTI was found positively correlated with MRP (*r* = 0.99) (Supplementary Table 2). In the present study, a higher mean seed yield was recorded in rainfed than in irrigated conditions. Therefore, DSI would not be a reliable criterion for selection when mean values under stress condition is higher than that of normal irrigated condition. Better performance of CDLs and their hybrids under rainfed condition compared to irrigated plots attributed to excessive lodging of plants in irrigated conditions. Although the level of this lodging in CDLs and hybrids is much less than what normally happens in *B. carinata* after irrigation or rainfall. Therefore, DTI and MRP were found to be better predictors of Y_*IR*_ and Y_*RF*_ than TOL and DSI. This study is in accordance with previous reports suggesting DTI as a better parameter for identifying drought-tolerant genotypes in mungbean (Fernandez, 1992), corn (Shirinzadeh et al., 2010), wheat (Farshadfar et al., 2012), chickpea (Pour-Siahbidi and Pour-Aboughadareh, 2013), durum wheat (Mohammadi, 2016), and barley (Khalili et al., 2016). Mahajan et al. (2018) also reported that DTI is a more desirable index for stress tolerance than SSI in rice under moisture deficit stress.

The test hybrids expressed improved WUE as compared to parental CDLs. Overall, the mean of seed yield (kg/ha) and WUE of parental CDLs and hybrids was recorded higher under rainfed condition than in irrigated conditions (Figure 1), indicating the presence of putatively different loci/genomic segments in the CDLs for improved WUE and yield heterosis. Expression of overdominance and/or complementation of favourable alleles in hybrids appear to be the reason for improved WUE and established genetic elite in the hybrids.

Higher MRP and DTI confirmed better tolerance/adaptation of hybrids to moisture deficit stress over the parental CDLs for most of the traits under study (Figure 2). Hybrids expressing DTI value of >1, indicating their superiority under both moisture-stress and non-stress conditions. Lower DTI value, on the other hand, did not precisely explain susceptibility to moisture deficit stress conditions as mean yield under rainfed was higher than irrigated in present study. CDLs carry introgressions from *B. carinata* lodged after irrigation or rain and, thus, lead to a reduction in seed yield. In general, higher MRP of hybrids than CDLs indicates better performance of hybrids under rainfed and irrigated conditions. Based on higher DTI and MRP, highly productive and drought-tolerant hybrids, *viz*., CDL102 × CDL186, CDL102 × CDL25, CDL102 × CDL89, CDL102 × CDL106, CDL161 × CDL128, CDL161 × CDL141, CDL161 × CDL186, CDL161 × CDL101, CDL128 × CDL141, CDL186 × CDL121, CDL186 × CDL101, CDL182 × CDL121, CDL121 × CDL25, CDL101 × CDL104, CDL101 × CDL106, CDL25 × CDL89, CDL25 × CDL104, CDL89 × CDL104, and CDL104 × CDL106 were identified (Table 8). These hybrids can be commercially exploited by converting the parental CDLs into CMS or restorer lines based on their seed production suitability. Hence, hybrid vigour expressed under moisture deficit stress conditions is well demonstrated through the deployment of *B. carinata*-derived *B. juncea* lines.

## Conclusion

In the present study, the availability of larger SCA variance than GCA variance for seed yield and its contributing traits under both water deficit and water sufficient conditions indicated a preponderance of dominance gene action. It suggests the manifestation of heterosis in *B. carinata*-derived *B. juncea* lines under moisture deficit stress conditions. The predominance of additive gene effects for traits like siliqua length and seeds per siliqua suggested the involvement of genetic and G × E interactions for these traits. Hybrids developed by involving CDLs with desirable SCA effects, higher heterobeltiosis, and better WUE exhibit overdominance and/or complement favourable alleles among different CDLs for expression of exploitable heterosis in hybrids. Many of the *B. carinata-*derived *B. juncea* lines that were identified in this study were heterotic and can be exploited for the development of commercial hybrids by converting them to cytoplasmic male sterile and/or restorer lines. CDLs, on the other hand, can also be used to recover desirable recombinants that would help in hastening the *Brassica* improvement programmes for the drought-prone rainfed areas. Material and information emanating from this study shall open new vistas for research involving related species for interspecific hybridization among *Brassica* species in creating novel genetic variability and deploying them for the development of high yielding hybrids with better WUE and, thus, overcoming the yield barriers in drought-prone regions.

## Data Availability Statement

The original contributions presented in the study are included in the article/Supplementary Material, further inquiries can be directed to the corresponding author/s.

## Author Contributions

OL conducted the field trials, generated crosses, analysed the data, and prepared the draft manuscript. RS conducted the trial, recorded observations, and maintained the CDLs. PV helped in data recording and analysis. PK and CP helped in conducting field trials, recording observations, and data analysis. JN helped in generating test hybrids. DY supervised the research. VC guided the research and edited the manuscript. NS developed the CDLs, conceptualized and supervised the research, analysed the data, and wrote the manuscript. All the authors read the manuscript and agreed with its content.

## Conflict of Interest

The authors declare that the research was conducted in the absence of any commercial or financial relationships that could be construed as a potential conflict of interest.

## Publisher’s Note

All claims expressed in this article are solely those of the authors and do not necessarily represent those of their affiliated organizations, or those of the publisher, the editors and the reviewers. Any product that may be evaluated in this article, or claim that may be made by its manufacturer, is not guaranteed or endorsed by the publisher.
