# Supplementary material for: Deployment of Brassica carinata A. Braun Derived Brassica juncea (L.) Czern. Lines for Improving Heterosis and Water Use Efficiency Under Water Deficit Stress Conditions
Source: Front Plant Sci. 2021 Nov 25;12:765645. doi: 10.3389/fpls.2021.765645 (PMC8655733; doi:10.3389/fpls.2021.765645)
Supplement: Supplementary file 1 [file Data_Sheet_1.docx]

Supplementary Table 1. Pedigree and morphological characteristics of *B. carinata* derived *B. juncea* lines (CDLs) along with their parents

| **Sr. No** | **CDL No.** | **Cross/ Pedigree** | **Plant height** | **Number of primary branches** | **Number of secondary branches** | **Siliqua length (cm)** | **1,000-seed weight (g)** | **Days to 50% flowering** | **Days to maturity** |
| --- | --- | --- | --- | --- | --- | --- | --- | --- | --- |
| 1 | CDL102 | DRMRIJ 31/ BC-4// DRMRIJ 31/ BC-4 | 205.0 | 4.4 | 9.2 | 6.2 | 5.8 | 42.0 | 151.0 |
| 2 | CDL161 | DRMRIJ 31/ BC-12// DRMRIJ 31/ BC-12 | 221.6 | 5.2 | 9.0 | 4.6 | 4.5 | 64.0 | 150.0 |
| 3 | CDL128 | DRMRIJ 31/ BC-4// DRMRIJ 31/ BC-4 | 208.8 | 4.2 | 8.4 | 4.6 | 4.7 | 52.0 | 154.0 |
| 4 | CDL141 | DRMRIJ 31/ BC-4// DRMRIJ 31/ BC-4 | 211.4 | 4.8 | 10.6 | 4.9 | 4.5 | 55.0 | 157.0 |
| 5 | CDL112 | DRMRIJ 31/ BC-4// DRMRIJ 31/ BC-4 | 237.4 | 4.4 | 10.2 | 4.3 | 5.1 | 65.0 | 153.0 |
| 6 | CDL186 | Pusa Mustard 30/ BC-4// Pusa Mustard 30/ BC-4 | 219.0 | 5.8 | 7.2 | 3.7 | 3.8 | 59.0 | 157.0 |
| 7 | CDL182 | Pusa Mustard 30/ BC-4// Pusa Mustard 30/ BC-4 | 221.2 | 4.2 | 13.6 | 4.7 | 5.0 | 47.0 | 152.0 |
| 8 | CDL121 | DRMRIJ 31/ BC-4// DRMRIJ 31/ BC-4 | 209.0 | 4.0 | 5.4 | 4.4 | 5.2 | 54.0 | 155.0 |
| 9 | CDL103 | DRMRIJ 31/ BC-4// DRMRIJ 31/ BC-4 | 205.4 | 4.0 | 6.4 | 4.9 | 6.3 | 47.0 | 151.0 |
| 10 | CDL101 | DRMRIJ 31/ BC-4// DRMRIJ 31/ BC-4 | 210.2 | 5.6 | 12.4 | 5.3 | 4.5 | 55.0 | 154.0 |
| 11 | CDL25 | Pusa Agrani/ BC-5// Pusa Agrani/ BC-5 | 206.2 | 4.8 | 14.0 | 3.6 | 4.1 | 46.0 | 156.0 |
| 12 | CDL89 | DRMRIJ 31/ BC-4// DRMRIJ 31/ BC-4 | 198.4 | 5.8 | 13.8 | 4.4 | 6.0 | 61.0 | 158.0 |
| 13 | CDL104 | DRMRIJ 31/ BC-4// DRMRIJ 31/ BC-4 | 202.8 | 4.0 | 11.0 | 5.7 | 5.1 | 53.0 | 153.0 |
| 14 | CDL105 | DRMRIJ 31/ BC-4// DRMRIJ 31/ BC-4 | 203.0 | 4.2 | 7.0 | 5.9 | 5.4 | 40.0 | 154.0 |
| 15 | CDL106 | DRMRIJ 31/ BC-4// DRMRIJ 31/ BC-4 | 220.2 | 4.0 | 10.0 | 5.4 | 6.2 | 48.0 | 154.0 |
| 16 | DRMRIJ 31 | *B. juncea* genotype | 202.5 | 4.6 | 8.6 | 4.2 | 5.5 | 49.5 | 158.0 |
| 17 | Pusa Mustard 30 | *B. juncea* genotype | 214.7 | 4.3 | 9.3 | 4.0 | 5.9 | 51.8 | 156.3 |
| 18 | Pusa Agrani | *B. juncea* genotype | 185.4 | 4.2 | 8.2 | 4.1 | 5.0 | 41.3 | 148.8 |
| 19 | BC-4* | *B. carinata* accession | 250.2 | 9.8 | 20.2 | 3.9 | 1.9 | 82.0 | 169.0 |
| 20 | BC-5* | *B. carinata* accession | 254.6 | 7.0 | 18.0 | 3.8 | 1.8 | 80.0 | 168.0 |
| 21 | BC-12* | *B. carinata* accession | 249.2 | 8.0 | 16.0 | 3.9 | 2.1 | 79.0 | 170.0 |

*: *B. carinata* genotypes, when compared to *B. juncea* genotypes and CDLs, have tall plant stature, profuse branching, small seed size and takes longer to flower and mature.

Supplementary Table 2. Correlation coefficients between water use efficiency, drought tolerance indices and seed yield performance in parents and their hybrids under rainfed and irrigated conditions

|  | Y_RF_ | Y_IR_ | WUE_RF_ | WUE_IR_ | DSI | DTI | TOL | MRP |
| --- | --- | --- | --- | --- | --- | --- | --- | --- |
| Y_IR_ | 0.65** |  |  |  |  |  |  |  |
| WUE_RF_ | 0.99** | 0.65** |  |  |  |  |  |  |
| WUE_IR_ | 0.65** | 0.99** | 0.65** |  |  |  |  |  |
| DSI | 0.49** | -0.33 | 0.49** | -0.33 |  |  |  |  |
| DTI | 0.90** | 0.89** | 0.90** | 0.89** | 0.10 |  |  |  |
| TOL | 0.54** | -0.29 | 0.54** | -0.29 | 0.98** | 0.16 |  |  |
| MRP | 0.92** | 0.90** | 0.92** | 0.90** | 0.11 | 0.99** | 0.16* |  |

** indicate significant at 1 % level

Y_RF_ = Seed yield under rainfed condition; Y_IR_ = Seed yield under irrigated condition; WUE_IR_ = water use efficiency under irrigated condition; WUE_RF_ = water use efficiency under rainfed condition; DSI = Drought susceptibility index; DTI = drought tolerance index; MRP =mean relative performance and TOL = tolerance index


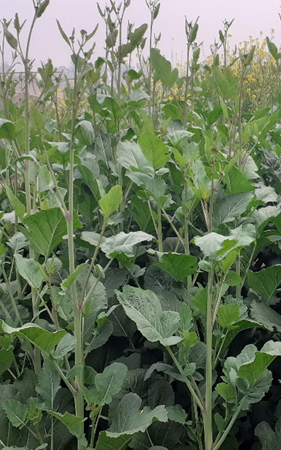
Supplementary Figure 1. Morphological characteristics of *B. carinata* derived *B. juncea* lines along with their parents. (a) *B. juncea* variety DRMRIJ 31; (b) *B. carinata* derived lines in DRMRIJ 31 background; and (c) *B. carinata* accession BC-4


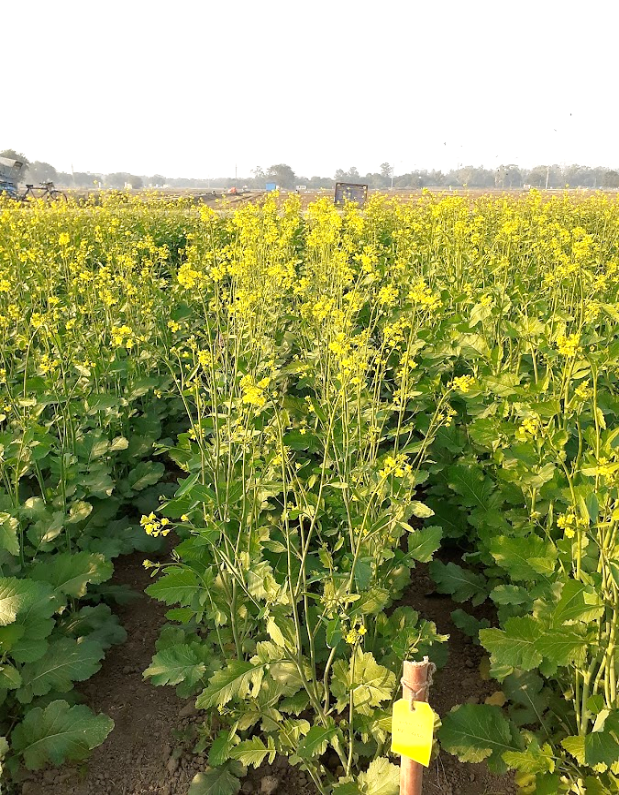

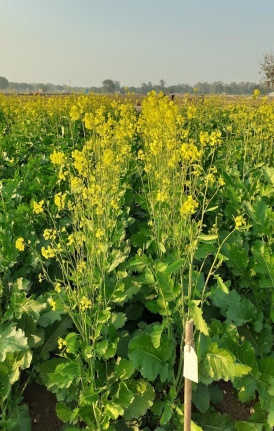


**(a)**

**(c)**

**(b)**

Supplementary Figure 2. Cytology of *B. carinata* derived *B. juncea* lines (CDLs) along with their parents. Diakinesis in (a) *B. juncea* variety DRMRIJ 31 (18 II; 2n=36); (b) CDLs (18 II; 2n=36); and (c) *B. carinata* accession (17 II; 2n=34).


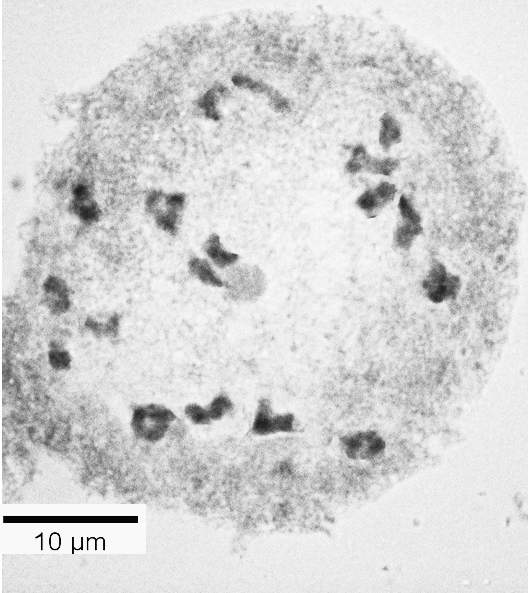

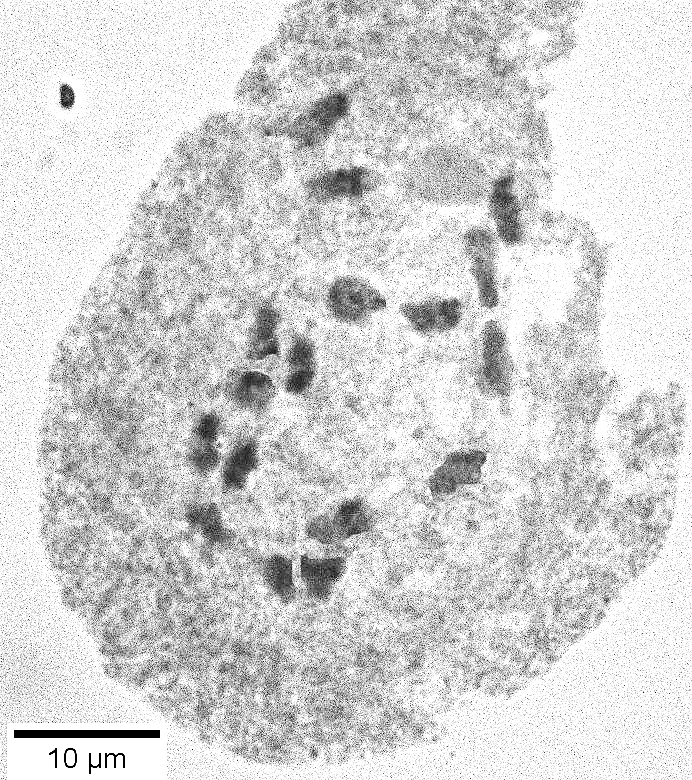


**(c)**


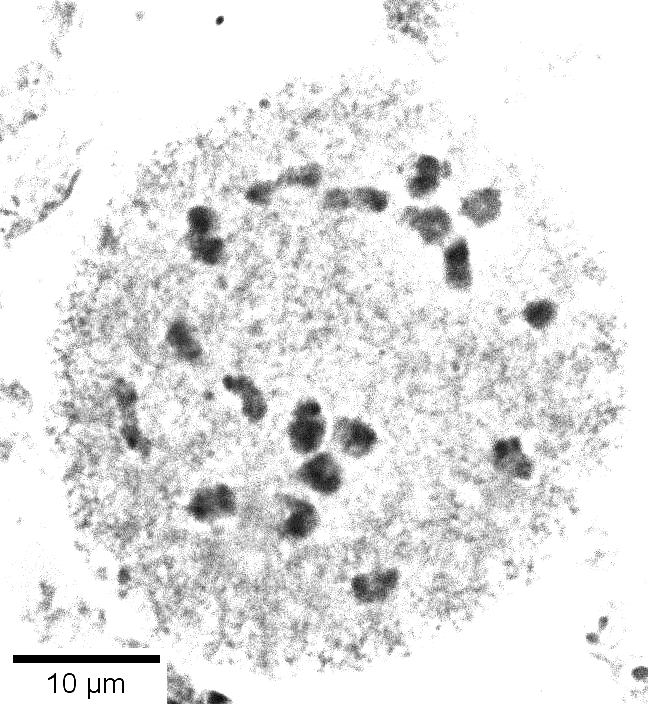


**(b)**

**(a)**

Supplementary Figure 3**.** Maximum and minimum temperatures and rainfall (mm) recorded during 2019-20 crop seasons

Vegetative stage

Flowering & siliqua development

Siliqua formation in secondary branches and seed filling

Seed filling to maturity

Maturity

Supplementary Figure 4. Notched box plots showing difference for seed yield contributing traits under rainfed and irrigated conditions


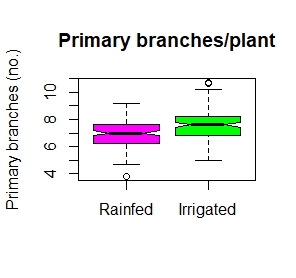

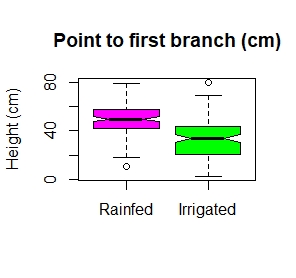

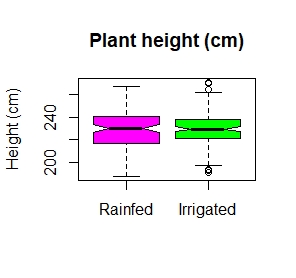

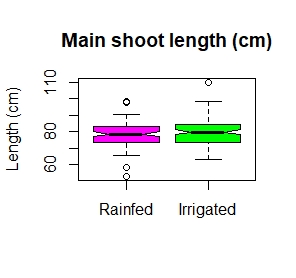

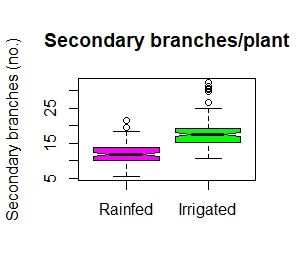

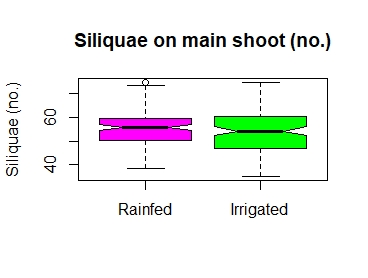

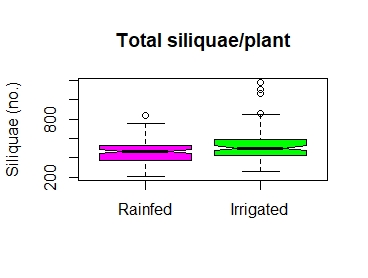

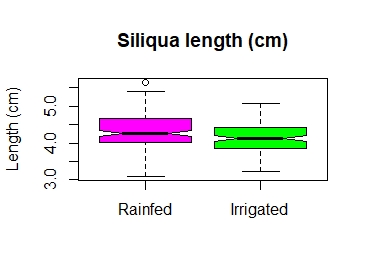

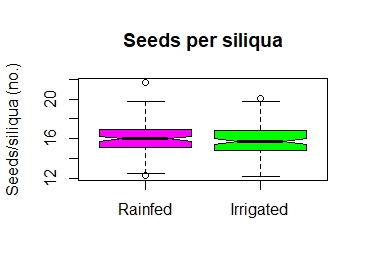

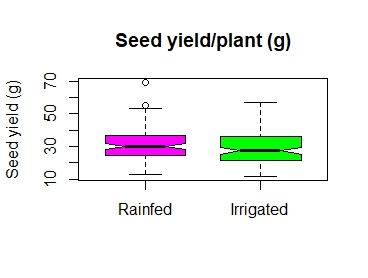

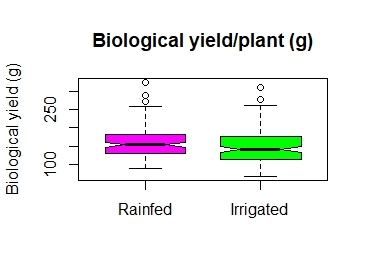

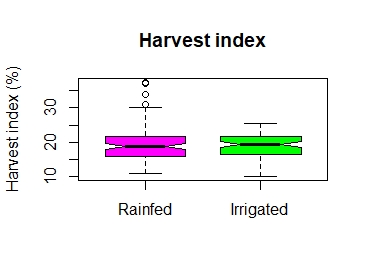

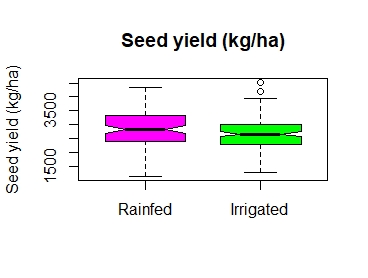

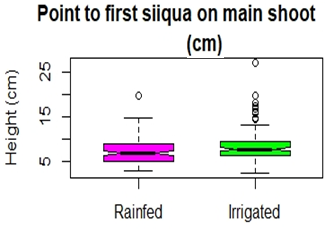

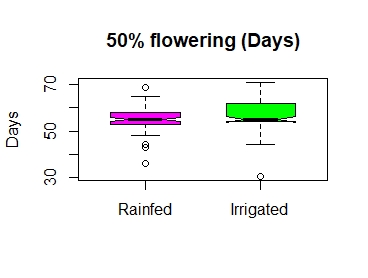

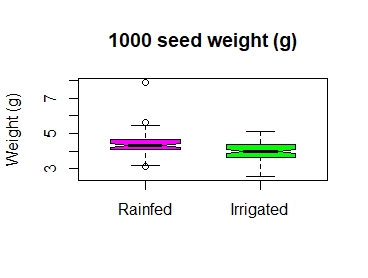

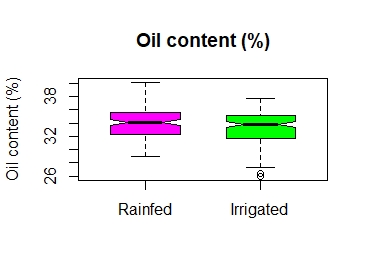


*Box edges represent upper and lower quartile, with median value shown as a bold line in the middle of the box. Whiskers represent 1.5 times the quartile of the data. Individuals falling outside the range of the whiskers shown as open dots.
